# Major transcriptome re-organisation and abrupt changes in signalling, cell cycle and chromatin regulation at neural differentiation *in vivo*

**DOI:** 10.1242/dev.112623

**Published:** 2014-08

**Authors:** Isabel Olivera-Martinez, Nick Schurch, Roman A. Li, Junfang Song, Pamela A. Halley, Raman M. Das, Dave W. Burt, Geoffrey J. Barton, Kate G. Storey

**Affiliations:** 1 Division of Cell and Developmental Biology, College of Life Sciences, University of Dundee, Dow Street, Dundee DD1 5EH, UK; 2 Division of Computational Biology, College of Life Sciences, University of Dundee, Dow Street, Dundee DD1 5EH, UK; 3 Department of Genomics and Genetics, The Roslin Institute and Royal (Dick) School of Veterinary Studies, University of Edinburgh, Edinburgh EH25 9RG, UK

**Keywords:** Neural differentiation, Transcriptome, Cell cycle, FGF signalling, Chromatin, Chick embryo

## Abstract

Here, we exploit the spatial separation of temporal events of neural differentiation in the elongating chick body axis to provide the first analysis of transcriptome change in progressively more differentiated neural cell populations *in vivo*. Microarray data, validated against direct RNA sequencing, identified: (1) a gene cohort characteristic of the multi-potent stem zone epiblast, which contains neuro-mesodermal progenitors that progressively generate the spinal cord; (2) a major transcriptome re-organisation as cells then adopt a neural fate; and (3) increasing diversity as neural patterning and neuron production begin. Focussing on the transition from multi-potent to neural state cells, we capture changes in major signalling pathways, uncover novel Wnt and Notch signalling dynamics, and implicate new pathways (mevalonate pathway/steroid biogenesis and TGFβ). This analysis further predicts changes in cellular processes, cell cycle, RNA-processing and protein turnover as cells acquire neural fate. We show that these changes are conserved across species and provide biological evidence for reduced proteasome efficiency and a novel lengthening of S phase. This latter step may provide time for epigenetic events to mediate large-scale transcriptome re-organisation; consistent with this, we uncover simultaneous downregulation of major chromatin modifiers as the neural programme is established. We further demonstrate that transcription of one such gene, *HDAC1*, is dependent on FGF signalling, making a novel link between signals that control neural differentiation and transcription of a core regulator of chromatin organisation. Our work implicates new signalling pathways and dynamics, cellular processes and epigenetic modifiers in neural differentiation *in vivo*, identifying multiple new potential cellular and molecular mechanisms that direct differentiation.

## INTRODUCTION

Important mechanisms that regulate vertebrate neural differentiation have been identified, such as the lateral inhibition controlling neuron production (Chitnis et al., 1995; Henrique et al., 1995). However, we know less about the cellular and molecular mechanisms that direct onset and progression of neural differentiation, and we lack a genome-wide analysis of key steps in this process in the embryo. Transcriptome analyses of neural differentiation have been carried out following selection of neural progenitors *in vivo* and by analysing differentiating ES cells (Li et al., 1998; Aubert et al., 2003; Abranches et al., 2009; Wu et al., 2010). However, these data are either confined to the neural progenitor cell state or *in vitro* represent a broader range of differentiation states (and cell types) at each time point than may be present at one time and place in the embryonic neural axis.

**Fig. 1.**
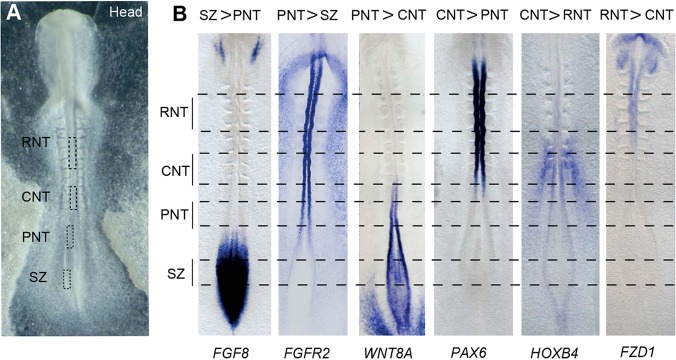
**Definition of distinct cell populations in the elongating neural axis.** (A) Definition of distinct cell populations along the stage 10 neural axis: stem zone (SZ), epiblast adjacent to the node/rostral primitive streak, also known as the caudal lateral epiblast; preneural tube (PNT), the open neural plate rostral to the node and two presumptive somites away from the last somite formed (somite I); caudal neural tube (CNT), the neural tube next to the last three formed somites (I to III); rostral neural tube (RNT), the neural tube flanked by somites 2 to 4. (B) Examples of known genes differentially expressed in each comparison made along the neural axis. Scale bar: 100 µm.

The spinal cord is generated progressively as cells leave the caudal region of the elongating body axis (Wilson et al., 2009), such that the temporal steps of neural differentiation become spatially separated along the head to tail axis. At key stages, it is therefore possible to isolate near-adjacent cell populations from the same embryo in distinct differentiation states (Fig. 1A). Cells in the caudal lateral epiblast adjacent to the primitive streak [also known as the stem zone (SZ) in the chick] (Wilson et al., 2009) express both early neural and mesodermal genes (Delfino-Machín et al., 2005), and there is evidence in the mouse that this cell population includes axial stem cells (Tzouanacou et al., 2009; Wilson et al., 2009). Other cells in the stem zone will gastrulate to form the paraxial mesoderm or remain in the epiblast cell sheet and become neural progenitors (Delfino-Machín et al., 2005). These latter cells form a new region called the preneural tube (PNT), which is flanked by unsegmented presomitic mesoderm; this represents an early neural progenitor state that can be induced by FGF signalling to revert back to a multi-potent SZ state (Diez del Corral et al., 2002). Cells in the PNT also undergo morphogenetic movements to close the neural tube. Rostral to this, the closed caudal neural tube (CNT) is flanked by somites and is an early site of co-expression of all three Sox1B genes, which are characteristic of neural progenitors (Delfino-Machín et al., 2005; Stavridis et al., 2010), and of key ventral patterning genes (Diez del Corral et al., 2003). The CNT contains the first few neurons and exposure to FGF cannot revert this tissue to a multi-potent SZ state (Diez del Corral et al., 2002). The transition from the PNT to the CNT thus involves commitment to a neural fate and we have demonstrated that this is regulated by a switch from FGF to retinoid signalling (Diez del Corral et al., 2003; Stavridis et al., 2010). More advanced neuroepithelium is then located in more rostral neural tube (RNT), in which neuronal differentiation is ongoing and dorsoventral pattern is refined. Here, we use the Affymetrix GeneChip chicken genome microarray to compare the transcriptomes of these spatially distinct cell populations from the elongating neural axis.

## RESULTS

### Defining tissues at distinct phases of neural differentiation

To identify key transcriptome changes at distinct phases of neural differentiation, we microdissected discrete cell populations, the SZ, PNT, CNT and RNT (Fig. 1A), from along the length of the chick neural axis at a single stage: HH stage 10 (ten somites) (Hamburger and Hamilton, 1951). Samples of each cell population were pooled separately and processed for screening the Affymetrix chick genome chip as described in the Materials and Methods. To confirm the identity of these cell populations, gene lists generated by comparison of neighbouring cell populations using linear model analysis for significantly differentially expressed genes (*P*<0.046) (supplementary material Table S1) were assessed for expression of 80 genes known to be non-ubiquitously expressed along the neural axis. All genes analysed were enriched in the predicted cell populations (Fig. 1B; supplementary material Table S2). Cloning and whole-mount *in situ* hybridisation was also used to validate the expression patterns of 25 genes not previously known to be differentially expressed along the neural axis. The expression patterns of all these genes were as predicted by the microarray (supplementary material Fig. S1; see below), further validating its representation of these transcriptomes. Annotation of the chick genome is, however, incomplete. Several novel algorithms were therefore developed to assign new annotations for microarray probe-sets (see Materials and Methods): of the 1678 microarray probe-sets for which we identified new annotations, 93% were associated with genes already represented in our highly significant gene lists. This suggests that the majority of differentially expressed genes in these tissues will already be captured by the microarray data. The chick embryo exhibits early sex-specific differences (Zhang et al., 2010; Zhao et al., 2010) and we therefore also controlled for potential sex bias that might appear despite sample pooling by comparison with a comprehensive list of cell-autonomous sex identity (CASI) genes (Zhao et al., 2010). This indicated no strong sex-linked bias in our datasets (see methods in the supplementary material).

Finally, to examine the dependence of our conclusions on the technological method used to probe the transcriptome, we sequenced PNT tissue with an alternative technology, Helicos Bio Direct RNA Sequencing (DRS) (Ozsolak and Milos, 2011). A direct comparison of 5178 ensembl genes with measured expression in both the DRS and microarray datasets revealed a reasonable correlation (Pearson correlation coefficient, R=0.68) between these two samples (see supplementary material Fig. S2 and methods in the supplementary material). To determine whether this largely reflects highly expressed housekeeping genes, we compared DRS and microarray data for the top 20 probe-sets (14 genes) changing between the PNT and the CNT. The chicken genome annotation is poor with regard to 3′UTRs, and many DRS reads were observed downstream of the limits of existing gene annotations. This prompted us to re-annotate 12 of these genes. This was carried out by integrating data across multiple technologies [DRS, publically available illumina RNA seq chicken data (SRP007412 GSE30352) and chicken EST data] and resulted in re-positioning 3′UTRs by up to 7 kb (Schurch et al., 2014). Following re-annotation, the correlation between PNT microarray data for the probe-sets and the DRS PNT signal for these genes is R=0.71, which compares well with previous comparisons between RNA Seq and microarray technologies (Sultan et al., 2008; Bradford et al., 2010). This suggests that the global correlation is not dominated by housekeeping genes or by poor annotation of 3′UTRs, and demonstrates the robustness of the microarray measurements.

### Neural differentiation onset involves major transcriptome re-organisation

Comparison of gene expression in tissues in the order in which differentiation proceeds along the neural axis (SZ to PNT, PNT to CNT, CNT to RNT) revealed that the greatest change takes place as cells transit from the PNT to the CNT (Table 1; supplementary material Table S1). This transition has the largest number of genes (593) showing significant regulatory changes, and identifies a localised and dramatic change in the transcriptome. Furthermore, whereas the other transitions show an approximate parity between upregulated and downregulated genes, the transition from the PNT to the CNT shows a significant imbalance, with ∼64% of the identified significantly regulated genes being downregulated. These marked differences suggest that the gradual change in the transcriptome, as prospective neural progenitor cells leave the SZ and enter the PNT, is then punctuated by a major re-organisation event, indicative of a fundamental change in cell state. This step happens in a region of the neural axis that corresponds to two to three somites in length and, as chick somites form here every 1.5 h, this suggests that this event is rapid, taking place within a 4.5 h period.

**Table 1.**
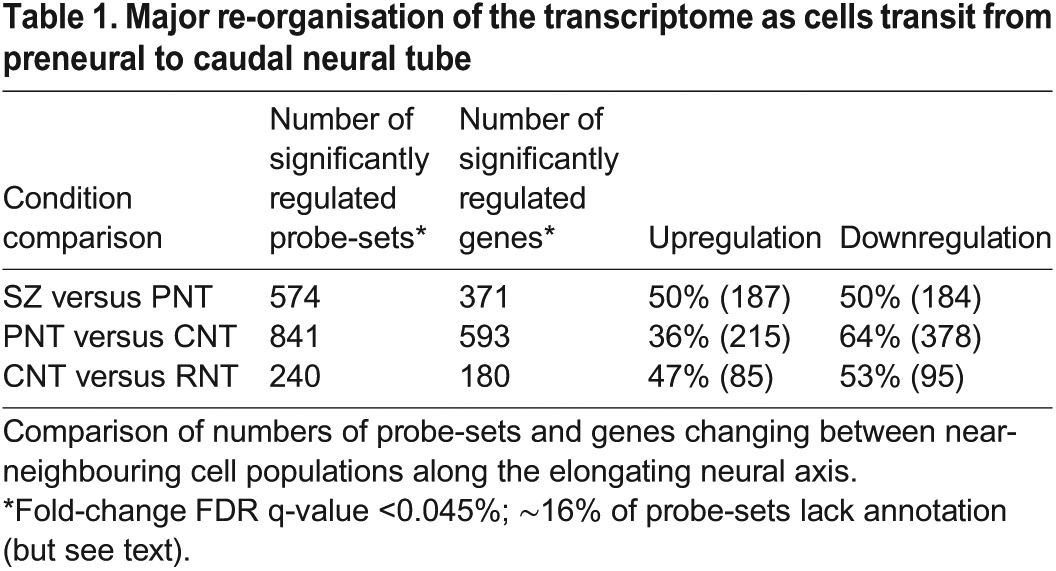
**Major re-organisation of the transcriptome as cells transit from preneural to caudal neural tube**

**Fig. 2.**
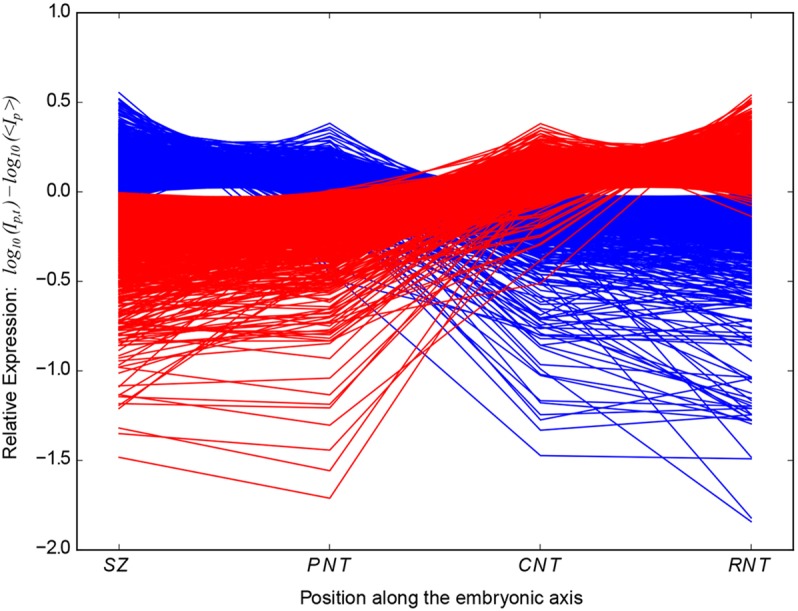
**Major transcriptome re-organisation depicted by plotting probe-set intensities for distinct cell populations along the neural axis.** The mean-scaled relative log_10_(expression) profiles along the developing embryonic axis of all 3178 probe-sets that show significant log_2_(fold change) between pooled SZ+PNT and CNT+RNT tissues. I_p,t_ is the intensity of probe-set ‘p’ in tissue type ‘t’ averaged across all replicates of the tissue. 〈I_p_〉 is the intensity of probe-set ‘p’ averaged across all replicates of all tissues. Blue and red lines show probe-sets that are upregulated and downregulated, respectively, in the CNT relative to the PNT. Large negative values indicate very low-level detections and are likely to correlate with an absence of gene expression.

As cells in the PNT, but not the CNT, can revert to SZ character (Diez del Corral et al., 2002; Olivera-Martinez and Storey, 2007), this suggests that SZ and PNT cells are within a spectrum of the multi-potent neural/mesodermal cell state and that final restriction to neural fate takes place as cells transit from the PNT to the CNT cell state. Similarly, CNT and RNT cells appear in a continuum of neural differentiation and there is no overall change in transcriptome size from the CNT to the RNT (Table 1). We therefore performed an additional Linear Model analysis by combining the (SZ+PNT) replicates and the (CNT+RNT) replicates, to identify genes whose expression change across the PNT to CNT switch is significantly greater than their expression changes between the SZ and the PNT or between the CNT and the RNT. This identified 3178 significantly regulated probe-sets, comprising 2116 unique genes (and 587 probe-sets with no identified gene association, but see Materials and Methods). This corresponds to ∼10% of the chicken genome and, as shown above, the majority of genes (1230, 58%) are downregulated (Fig. 2).

### Genes/KEGG pathways defining distinct cell populations along the neural axis

To identify major changes taking place between each cell population along the neural axis, we next analysed the top ∼50 significantly differentially expressed genes between each comparison and also applied gene set enrichment analysis of Kyoto Encyclopedia of Genes and Genomes (KEGG) pathways to all significantly regulated probe-sets (at >2σ, *P*<0.046) (supplementary material Table S1).

**Fig. 3.**
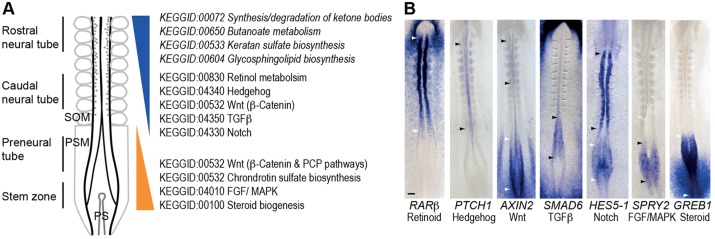
**Changes in signalling activity along the elongating neural axis.** (A) Changes in significantly regulated signalling pathways indicated by KEGG pathway analysis of the PNT versus the caudal neural tube CNT (additional KEGG terms identified in pooled SZ+PNT versus CNT+RNT analysis in italics). SOM, somites; PSM, presomitic mesoderm; PS, primitive streak. (B) Examples of signalling indicators for seven pathways exhibiting localised activity along the neural axis. Regions between arrowhead pairs (black or white) indicate sites of elevated signalling. Scale bar: 100 µm.

**Fig. 4.**
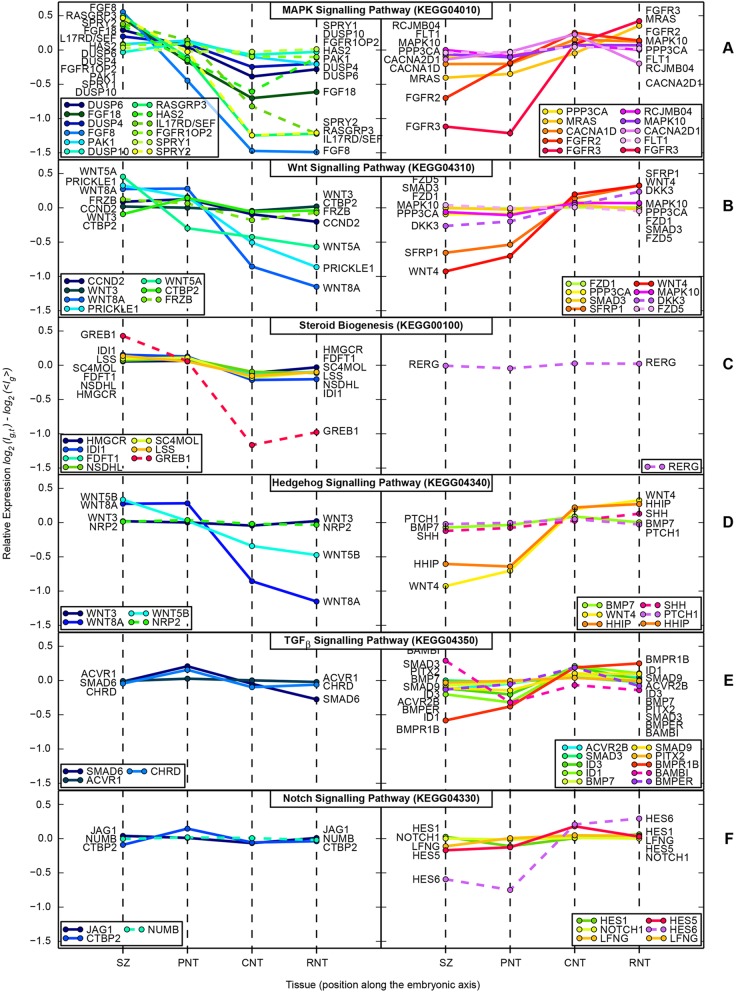
**KEGG pathway gene expression profiles along the neural axis.** (A-F) KEGG pathways whose gene annotations are significantly over-represented within the list of genes identified as significantly differentially expressed between SZ+PNT and CNT+RNT. Upper half of each panel shows expression profiles of genes upregulated; lower half shows genes downregulated across the ‘differentiation switch’. The value plotted on the *y*-axis is log_2_ relative expression, scaled to the mean expression of that gene across all tissues. This represents the fractional change, rather than the absolute change, in expression level across the switch; highly expressed genes may show statistically significant differential expression even for small changes in relative expression. Profiles shown with solid lines were annotated to the pathway within the ‘chicken.db’ R package (and thus represent the set that the enrichment test is performed against); those with dotted lines were manually assigned from the lists of statistically significantly regulated genes. (A) MAPK pathway; (B) Wnt pathways; (C) steroid biosynthesis pathway; (D) Hedgehog pathway (note that KEGG annotates some Wnt pathway genes to the Hedgehog pathway); (E) TGFβ pathway; (F) Notch pathway.

The genes most highly expressed in stem zone in comparison with preneural tube included multiple pathways that were newly implicated in the regulation of this cell population: (1) *GFRA1* (glial-derived nerve growth factor receptor), NGF-induced gene *EGR1/KROX24*, *UNC5B/NETRIN1* receptor and *EPHA1*, which are normally associated with cell adhesion and axon guidance; (2) steroid hormone signalling, indicated by elevated *GREB1* expression (gene regulated by oestrogen in breast cancer 1), a target of oestrogen and androgen receptors that also has 5′ binding sites for glucocorticoid and progesterone receptors (Rae et al., 2006; Deschenes et al., 2007) (validated by *in situ* hybridisation in supplementary material Fig. S1 and see below); (3) *PDGFR-alpha*, which is implicated in cell proliferation, survival and chemotaxis; and (4) *CD3-epsilon*, an immunoglobulin superfamily member and a constituent of the T cell receptor (Kuhns and Badgandi, 2012). KEGG analysis essentially confirmed the representation of two known signalling pathways, FGF/MAPK and Wnt (Fig. 3A,B; Fig. 4A,B). Consistent with co-expression of mesoderm and neural progenitor genes in the stem zone, representative transcription factors included mesodermal genes *T* (brachyury), *cNOT2* and *gNOT1*. Genes expressed highly in the PNT in comparison with the SZ are involved in a distinct set of signalling pathways: potassium and magnesium ion channel genes; fatty acid binding proteins (e.g. *FABP5* validated by *in situ* hybridisation in supplementary material Fig. S1) linked to PPARβ/δ and retinoid signalling; *VEGFR3*; and the first expression of Shh receptor *PTCH1* and associated ventral patterning transcription factors *NKX6.1* and *NKX6.2*.

Many of the most highly expressed genes in the PNT compared with the CNT were related to FGF and Wnt (canonical and PCP) pathways and often associated with Hox/Cdx transcription factors, while KEGG pathway analysis additionally identified MAPK and steroid biosynthesis (Fig. 3A,B; Fig. 4A,C; Table 2). Notable genes more strongly expressed in the CNT compared with the PNT mapped to Hedgehog, TGFβ and Notch signalling (Fig. 3A,B; Fig. 4D-F; Table 2), and included transcription factors associated with neurogenesis (*ZIC1*, *IRX1*, *HES6*, *NR2F2*, *DBX2*, *FOXD3*, *PITX2*, *PAX6*, *NKX6.2*) and cell adhesion/junction-associated genes (*L-CAM-like*, cadherin 20, integrin alpha6 receptor, *TMFM47 claudin-related*) (supplementary material Table S1).

**Table 2.**
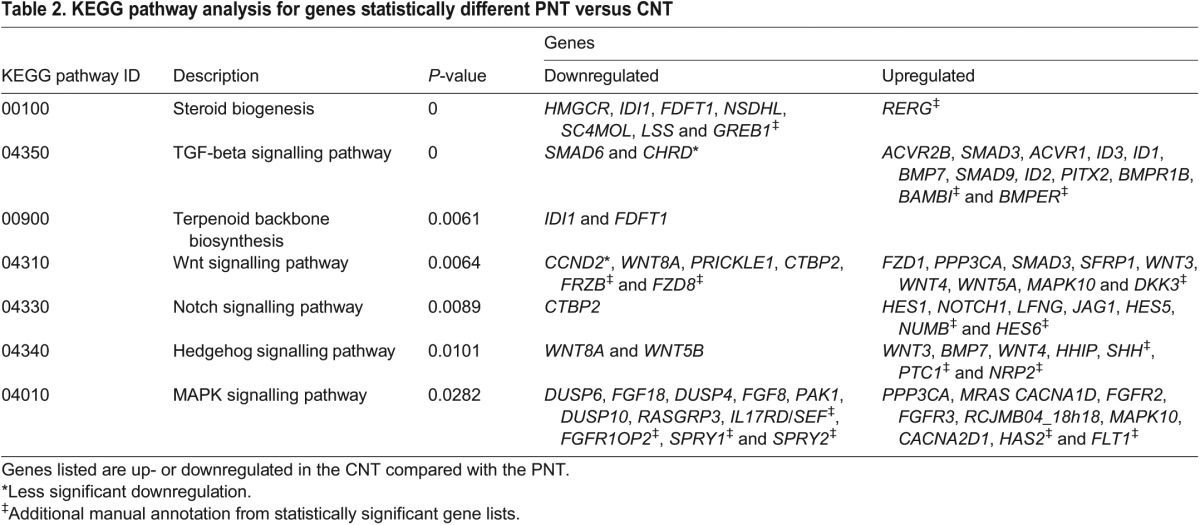
**KEGG pathway analysis for genes statistically different PNT versus CNT**

Noteworthy genes elevated in the CNT compared with the RNT included those encoding a calcium channel (*CACNA2D1*), a zinc transporter (*SLC39A8*) and a further solute carrier (*SLC7A9*). KEGG analysis produced increasing representation of ErbB, Wnt and MAPK pathways, as well as further neural patterning transcription factors such as *SOX18* and *EMX2* (supplementary material Table S1). The converse comparison revealed increased expression of extracellular matrix-associated genes, such as *ADAMTS3*, *COL9A3* (collagen type IX alpha 3), *FGB* (fibrinogen beta chain) and *TIMP3*; and of neuronal guidance and signalling molecules [e.g. *SST* (somatostatin), *PLXNC1*, *NTN3* (netrin 3)]. Finally, KEGG analysis based on the pooled comparison (SZ+PNT versus CNT+RNT) identified many of the same pathways as for PNT versus CNT, but added upregulated terms in the CNT+RNT set (Fig. 3A), including glycosphingolipids, the major glycan of the vertebrate nervous system associated with neuronal differentiation and myelination.

### Discrete changes in signalling activity punctuate neural differentiation progression

By focusing in detail on changes at the onset of neural differentiation captured in the pooled (SZ+PNT versus CNT+RNT) comparison, we found key transcriptional targets for many significantly altered pathways. Importantly, this identified novel and local changes in signalling activity along the neural axis (Table 2; Fig. 3A,B; Fig. 4A-F; supplementary material Table S1). Pathways known to be discretely active along the neural axis were indicated by expression of transcriptional targets of FGF/MAPK signalling, i.e. SPRY genes and DUSP genes (Minowada et al., 1999; Eblaghie et al., 2003; Lunn et al., 2007), by expression of the canonical Wnt signalling readout gene *Axin2* in the SZ/PNT, the sonic hedgehog receptor and transcriptional target *PTCH1*, *SHH* ligand and its antagonist *HHIP*, and the retinoid target genes *RARB* and *CYP26A* in the CNT/RNT (Table 2; Fig. 3B; Fig. 4). Novel signalling activity included the discrete expression of inhibitory TGFβ/BMP signalling intermediate SMAD6 (Park, 2005) in the SZ/PNT (Fig. 3B) and, conversely, the increased expression of BMP and TGFβ ligands, receptors, transducers and known target genes in the CNT/RNT. This indicates a sharp onset of TGFβ/BMP signalling as neural differentiation commences and identifies regulation of *SMAD6* transcription as a potential mediator of this signalling event (Table 2; Fig. 4E). In addition, expression analysis of the Notch signalling targets *HES1* and *HES5-1* (Fior and Henrique, 2005), although increased in the CNT/RNT as predicted, indicated a transient repression of Notch activity as cells form the PNT (Fig. 3B; Fig. 4F). Restriction of transcripts for planar cell polarity mediators (*WNT5A*, *WNT5B* and *PRICKLE1*) to the SZ/PNT, along with downregulation of mediators of canonical Wnt signalling and upregulation of secreted Wnt antagonists (*DKK3*, *SFRP1*) in the CNT/RNT, indicate a dramatic attenuation of both these Wnt pathways at the onset of differentiation (Table 2; Fig. 3B; Fig. 4B). Canonical Wnt signalling is then re-established in the RNT and these signalling dynamics are captured by the expression pattern of the target gene *AXIN2* (Fig. 3B).

Finally, KEGG analysis indicated increased expression in the PNT of six genes mediating steroid biogenesis, which are essentially members of the HMG-CoA reductase/mevalonate pathway (including HMGCR, the mevalonate pathway rate-limiting enzyme) that leads to cholesterol (and ergosterol) production (Table 2; supplementary material Table S3; Fig. 4C). Cholesterol can enter the cell membrane where it plays a role in post-translational modification of hedgehog proteins, is used to generate many steroids or can be stored in cytoplasmic lipid droplets (Willnow et al., 2007). The mevalonate pathway also generates dolichols, which are involved in protein glycosylation, and isoprenoids, which mediate protein prenylation and are required for protein association with internal membrane. Cloning and expression pattern analysis of five of these genes confirmed elevated levels in the SZ/PNT (Fig. 4C; supplementary material Fig. S1). Furthermore, levels of *GREB1*, a target of oestrogen and other steroid hormones (Rae et al., 2006; Deschenes et al., 2007) elevated in the SZ, were also increased in SZ+PNT versus CNT+RNT. Cloning and *in situ* hybridisation for *GREB1* confirmed discrete, high level expression in the SZ and the PNT, as well as in the emerging caudal paraxial mesoderm (Fig. 3B; Fig. 4C), implicating new hormone signalling pathways and cellular processes in the generation of the body axis.

### Transcription factor repertoire reflects the switch in signalling at differentiation onset

Representation of significantly regulated transcription factors (GO term 0030528) across the SZ+PNT versus CNT+RNT comparison (supplementary material Table S4) also reflects the shift from an FGF/MAPK orchestrated cell state: the majority of the SZ+PNT gene cohort were Hox/Cdx Ets and AP1 genes, many of which are known to be downstream of FGF/MAPK signalling, whereas CNT+RNT highly expressed genes included a greater range of transcription factor classes (homeobox, nuclear receptors, Forkhead, MADS-box, bHLH, Ets, LIM, bZip, paired box, zinc finger, E-Box, E2F, ETO domain-containing genes). Regional differences in Hox gene expression identified in the microarray are as predicted from known gene expression patterns at HH10 (e.g. Bel-Vialar et al., 2002; see supplementary material Table S5). The RNT corresponds to future caudal hindbrain and the CNT to rostral spinal cord, whereas cells in the SZ and the PNT will contribute extensively along the rostrocaudal extent of the spinal cord (Stern et al., 1991; Brown and Storey, 2000). Importantly, Hox gene boundaries are not fixed in caudal regions at this stage (Deschamps et al., 1999) and this is reflected in our data: cells in the CNT, PNT and SZ co-express genes from multiple Hox gene paralogous groups (4 to 7) (supplementary material Table S5; see *HOXB4* expression in the PNT and the CNT in Fig. 1B). Hox gene expression domains are resolved as development proceeds and it is clear that establishment of regional identity is linked to the differentiation process. Indeed many cells in the SZ and the PNT will become CNT cells and at this early stage differences between these regions largely reflect differentiation status.

### Changes in key cellular processes at differentiation onset

Comparison of pooled SZ+PNT with CNT+RNT additionally identified KEGG terms associated with key cellular processes. In particular, downregulated genes in the CNT+RNT pool included ten aminoacyl-tRNA biosynthesis-associated genes [all aminoacyl-tRNA synthetases (AARSs) that ligate specific amino acids to tRNAs] as well as numerous genes encoding proteasome subunit/catalytic proteins (supplementary material Table S6). Also of interest, although outside our significance threshold (*P*<0.14), are cell cycle genes, many of which are also downregulated (supplementary material Table S6). GO term analysis confirmed enrichment of tRNA metabolic processes as well as of genes involved in ribosomal RNA processing in the SZ+PNT (supplementary material Table S7), predicting a change/reduction in translation efficiency as neural differentiation is initiated. Further notable GO terms assigned to SZ+PNT map well to cell shape changes during gastrulation and neurulation, and, in CNT+RNT, to cellular processes underpinning neuronal differentiation (supplementary material Table S7).

### Biological evidence for predicted changes in cell cycle and protein turnover

**Fig. 5.**
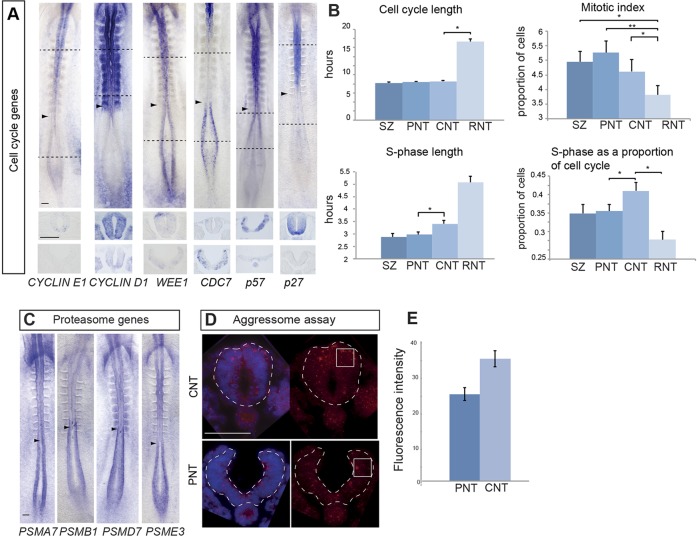
**Cell cycle and epigenetic gene expression patterns, and analysis of cell cycle parameters and proteasome efficiency.** (A) Whole-embryo mRNA *in situ* hybridisation for key cell cycle genes *cyclin E1*, *cyclin D1*, *WEE1* and *CDC7*, and the Cdk inhibitors *P57* and *P27* (black dashed lines indicate the position of transverse sections beneath each embryo). (B) Histograms for cell cycle time, mitotic index, S phase duration and S phase as a proportion of cell cycle time in each of the four regions assessed along the neural axis (*P<0.05; **P<0.001). CNT, caudal neural tube; PNT, preneural tube; RNT, rostral neural tube; SZ, stem zone. Data are mean±s.e.m. (C) Whole-embryo mRNA *in situ* hybridisation for four genes encoding proteasome component proteins predicted by microarray to be enriched in the SZ and the PNT (indicated with arrowheads) compared with the CNT. (D) Aggresomes identified by ProteoStat labelling kit (red) in the PNT and the CNT. (E) Quantification of fluorescence intensities measured in sections of each tissue in quadrants (white outlined boxes) (*n*=5 embryos, at least four sections for each region) and compared using an unpaired *t*-test, *P*<0.001. Data are mean±s.e.m. Scale bars: 100 µm.

To determine whether predicted local changes in cellular processes have functional consequences, we first focused on cell cycle parameters. Key cell cycle genes identified by KEGG analysis (supplementary material Table S6) were cloned [*CCNE1* (cyclin E1), *WEE1*, *DBF4*-binding partner *CDC7*] or had been previously characterised [*CCND1* (cyclin D1 (Lobjois et al., 2004)] and their mRNA expression patterns analysed (Fig. 5A). This identified reduced expression in the CNT of G1 entry cyclins *CCNE1* and *CCND2* (Lobjois et al., 2004), while *CCND1* is detected at low levels from the CNT increasing in the RNT (Fig. 5A). A transcriptional lull in *CDC7* [which encodes a protein required for initiation of replication origins throughout S phase (Jares et al., 2000)] was also strikingly apparent in the CNT (Fig. 5A). Further genes associated with DNA replication were also captured by KEGG analysis, including *ORC4L* and *ORC3L* (supplementary material Table S6). Transcripts for *WEE1*, which regulates entry into mitosis (Perry and Kornbluth, 2007), were detected with increasing levels in the CNT and the RNT, but were dorsally restricted, suggesting the existence of additional isoforms or of *WEE1*-like genes (Fig. 5A). We additionally characterised the expression of key Cdk inhibitors *P57* and *P27* as these may indicate where cell cycle exit is first possible along the forming neural axis; these were found to commence in the rostral-most PNT, increasing in the CNT/RNT (Fig. 5A). These expression patterns predict an increase in cell cycle length in the neural tube as CdkI levels determine entry into S phase. Consistent with this, we find that cell cycle time doubles from ∼8 h in the SZ to ∼16 h in the RNT (Fig. 5B) (see Wilcock et al., 2007) and the mitotic index is reduced (Fig. 5B). As cell cycle progression depends on rapid protein turnover, this might also reflect reduced proteasome efficiency predicted by downregulation of proteasome subunits in the CNT/RNT. Cloning and expression analysis of four proteasome genes identified in the microarray (supplementary material Table S6) confirmed higher levels of transcription in the SZ/PNT (Fig. 5C). Consistent with this, more aggresomes, which accumulate when the ubiquitin/proteasome machinery is less effective (Amijee et al., 2009), were found in CNT cells compared with PNT cells (Fig. 5D,E). These direct functional data therefore support *in silico* predictions of changes in cell cycle length and proteasome efficiency as neural differentiation commences.

The overall extension of the cell cycle in differentiating neuroepithelium must, however, be distinct from the effects of the transient transcriptional hiatus of the crucial S phase gene *CDC7* in the CNT, which would be predicted to slow down DNA replication. Comparison of cell cycle times and S phase length in each of the four regions of the neural axis revealed that although total cell cycle time is not longer between the PNT and the CNT, S phase does indeed lengthen (>30 min) (Fig. 5B) and occupies proportionally more of the cell cycle in the CNT (Fig. 5B). This supports the microarray prediction and suggests a new step, transient S phase extension, during the establishment of the neural differentiation programme.

### Conservation of cellular process changes on differentiation onset

Importantly, a similar cell state change with respect to downregulation of genes associated with cell cycle regulation and RNA processing, was detected in the transcriptome of whole mouse embryos at the onset of organogenesis (Mitiku and Baker, 2007). Our findings here localise this cell state change to a discrete cell population in the embryonic body axis. This downregulated gene cohort in the mouse was found to be analogous to 65 RNA processing and cell cycle genes downregulated in post-gastrula *Drosophila* embryos (Arbeitman et al., 2002; Mitiku and Baker, 2007). To evaluate the conservation of this phenomenon in the chick neural axis, we queried the Ensembl database for chicken gene orthologs of the 65 *Drosophila* genes. Probe-sets associated with 29 of these genes showed significant differential expression changes in at least one of our tissue comparisons. The probability of obtaining this result by chance is low (*P*=0.001; see methods in the supplementary material). The expression pattern of these genes along the neural axis falls broadly into two clusters (supplementary material Fig. S3), but indicates an overall pattern in which downregulation of conserved RNA processing and cell cycle gene expression is transient and correlated with the onset of differentiation.

### Transcriptional repression of key chromatin-modifying genes at differentiation onset

**Fig. 6.**
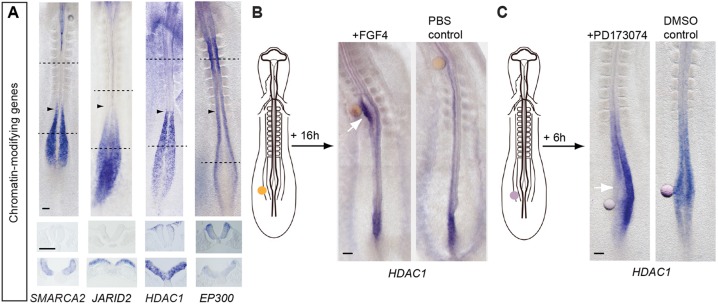
**Downregulation of genes encoding chromatin modifiers and *HDAC1* regulation by FGF signalling.** (A) Transcripts for key chromatin-modifying genes *SMARCA2*, *JARID2* and *HDAC1* are downregulated as neural differentiation begins, whereas expression of *EP300*, which counters the activity of *HDAC1*, increases (arrowheads indicate the most recently formed somite; black dashed lines indicate the position of transverse sections beneath each embryo). (B) FGF-presenting beads transplanted into the stem zone maintain local ectopic *HDAC1* transcription (white arrow). (C) Beads presenting the FGFR inhibitor PD173074 locally repress *HDAC1* transcription in the stem zone (white arrow). Scale bars: 100 µm.

It is possible that elongating S phase in the CNT allows time for epigenetic events to take place that facilitate large-scale transcriptome re-organisation. Consistent with this, we find a sharp downregulation of transcripts for genes encoding major chromatin-modifying proteins, including the ATP-dependent chromatin remodelling gene *SMARCA2/*Brahma homologue/BAF190B (Goodwin, 1997), the polycomb repressor complex (PRC)-associated gene *JARID2* (Landeira and Fisher, 2011) and the histone deacetylase *HDAC1*, which is complemented by upregulation of EP300, a key histone acetylase (identified in GO and/or KEGG analyses, and validated by cloning of these genes and mRNA expression analysis in the embryo (Fig. 6A).

### FGFR signalling promotes and maintains *HDAC1* transcription

To link such chromatin-modifying complexes to the signalling switch that regulates neural differentiation, we then tested whether transcription of *HDAC1*, which is associated with multiple repressor complexes (including polycomb), was regulated by FGF signalling. Heparin beads presenting either FGF8 or FGF4, or control PBS-washed beads were grafted beneath the stem zone epiblast of ten-somite stage embryos, which were then cultured for 16 h (Fig. 6B). This maintained FGF signalling in cells immediately adjacent to the bead and led to local ectopic expression of high-level *HDAC1* in the neural tube in response to FGF8 (15/20) and FGF4 (3/3) in comparison with control PBS-washed beads (0/6) (Fig. 6B). To determine whether FGF receptor signalling is required for maintenance of *HDAC1* transcription in stem zone epiblast, this region was grafted with beads presenting the small-molecule FGFR inhibitor PD173074 or vehicle DMSO-only control (Fig. 6C). Embryos were then cultured for 4-6 h during which beads remained within the endogenous *HDAC1* domain. This lead to local reduction of *HDAC1* transcripts in the presence of the FGFR antagonist (7/10) but not in DMSO controls (0/7) (Fig. 6C). FGFR signalling thus promotes and is required for *HDAC1* transcription at the caudal end of the embryo.

## DISCUSSION

This study captures transcriptome changes that take place as cells progress from the multi-potent stem zone/caudal lateral epiblast towards neural differentiation and subsequent patterning and neurogenesis in the elongating body axis. We identify a cohort of stem zone/CLE genes that newly connect this axial stem cell-containing tissue with molecules that regulate cell adhesion/guidance, T-cells and mevalonate/steroid/lipid signalling. Our data further uncover a rapid transcriptome switch as cells transit from multi-potent neuro-mesodermal potential to a neural cell state. This includes changes in known differentiation signals and identifies novel signalling dynamics and pathways, as well as conserved changes in fundamental cellular processes. Functional validation corroborated changes in protein turnover and cell cycle regulation, including an extension of S phase, which coincides with downregulation of multiple chromatin-modifying genes as the neural programme is established. We additionally link this phenomenon to the signalling switch that controls differentiation, demonstrating that transcription of one such gene, *HDAC1*, is regulated by FGF signalling. Further progress through neural development is captured by comparison of caudal and rostral neural tube transcriptomes, providing a resource for identification of genes associated with the later steps of patterning and neurogenesis.

Our analysis has focussed on the transcriptome switch that marks neural differentiation onset uncovered in this dataset. We have previously established that a switch from FGF/Wnt to retinoid signalling controls onset of differentiation genes in the embryonic body axis (Diez del Corral et al., 2003; Olivera-Martinez and Storey, 2007; Stavridis et al., 2010) and the transcriptome analysis here captures further novel signalling activities that characterise this step. These new insights include: sharp onset of TGFβ signalling (downregulation of key antagonist *SMAD6*) as neural differentiation commences; quiescence of Notch activity (discrete decrease in *Hes5-1*) in the PNT as cells transit from mutual (in the stem zone) to lateral inhibition (in the neural tube) modes of Notch signalling (Akai et al., 2005); dramatic downregulation of both planar cell polarity and canonical Wnt signalling pathways at neural differentiation onset; and localised activation of steroid signalling in the SZ/PNT, indicated by *GREB1*, the direct target of multiple steroid hormones. Defects in steroid biogenesis are associated with a range of human congenital developmental defects that include skeletal and facial deformities, as well as neural hypoplasia (Willnow et al., 2007). *GREB1* also mediates oestrogen-induced proliferation in mouse ES cells (Rae et al., 2005) and it will be important to investigate its regulation and function during body axis elongation. The SZ/PNT enrichment for transcripts of genes in the mevalonate pathway (which additionally generates short prenyl-group-containing lipids that target small GTPases to membranes where they mediate growth-promoting signalling) and for coenzyme Q (a soluble antioxidant that is also part of the respiratory chain in mitochondria) suggests metabolic changes associated with a shift away from rapid cell proliferation as differentiation begins. Indeed, a reduction in both lipid biogenesis and mitochondrial gene expression/oxidative phosphorylation has recently been linked to increased cell size (Miettinen et al., 2014), and here this would be further correlated with the onset of differentiation and increasing cell cycle length.

The slowing of the cell cycle was predicted by downregulation of key cell cycle and RNA processing genes as neural differentiation starts in the CNT. Investigation of changes in the whole-mouse embryo transcriptome at key developmental stages previously identified the specific downregulation of RNA processing and cell cycle genes at the onset of organogenesis at E8-8.5 (Mitiku and Baker, 2007). These authors suggested that transient reduction in RNA metabolism serves to clear the gene expression palate to allow new programmes of gene expression to be established. The identification of a cohort of downregulated RNA processing and cell cycle genes in chick, mouse and fly (Arbeitman et al., 2002) further reinforces this conserved feature of differentiation onset. In addition, transcriptome changes along the differentiating zebrafish paraxial mesoderm reveal a similar local re-organisation, which correlates with the onset of mesoderm differentiation that likewise includes genes mapping to GO terms Cell cycle/DNA metabolism (Ozbudak et al., 2010). In the fish embryo, RNA processing is upregulated rather than downregulated at this point, but this may reflect the more advanced differentiation cell state sampled in the fish. Intriguingly, in the chick, many of the downregulated RNA-processing genes were tRNA synthetases, raising the possibility that differentiation involves a change in tRNA abundance and codon use. This would alter translation efficiency and could confer preference for translation of specific mRNAs. Indeed, tRNA expression repertoire is reported to be tissue specific and varies in distinct cellular conditions (Dittmar et al., 2006).

The cell cycle is known to lengthen as cells begin to differentiate in some contexts, and cell cycle progression depends on rapid protein turnover; it is possible that reduced efficiency of this process, predicted by decreased transcription of proteasome subunit genes and validated by aggresome accumulation, contributes to cell cycle lengthening. More instrumental in cell cycle time extension, however, must be transcriptional onset of the CDK inhibitors *P27* and *P57*, and a transcriptional lull of a key initiator of DNA replication, *CDC7*, in the CNT. This latter event predicts slower progression through S phase and this is validated by our functional data. S phase elongation in the CNT and the gradual extension of the cell cycle as the neural programme is established may be indicative of the time taken for epigenetic changes to take place as cells transit the cell cycle during G1, when origins of replication are licensed, and during S phase, as chromatin is re-assembled after the replication fork.

The operation of widespread epigenetic changes directed by the signalling switch from FGF/WNT to retinoic acid may account for the coordinated onset of neural differentiation genes in the CNT. Evidence supporting coincident regulation by epigenetic mechanisms is manifest here by abrupt downregulation of key chromatin modifiers *SMARCA2*, *JARID2* and *HDAC1* in the CNT. SMARCA2 is part of the ATP-dependent nucleosome-positioning complex SNF/SWI, which is often associated with transcriptional activation (Lessard et al., 2007). Histone deacetylases remove histone acetyl groups, resulting in chromatin compaction, and HDAC1 is associated with repression complexes, including the PRC (van der Vlag and Otte, 1999; Martin et al., 2013), which also compacts chromatin. In ES cells, many known PRC targets are differentiation genes and PRC target specificity is conferred by cell-type-specific transcription factors such as *JARID2*. Consistent with the operation of coordinated widespread epigenetic mechanisms at the onset of neural differentiation, we show here that *HDAC1* transcription is promoted and maintained by FGF signalling in the SZ/PNT, making a novel link between the signalling switch that controls differentiation and a key mediator of chromatin state. We have also shown recently in the mouse embryo that FGF promotes compaction around neural differentiation genes in stem zone cells (Patel et al., 2013). Together, these findings connect FGF signalling to molecular machinery that orchestrates chromatin organisation around neural differentiation genes and indicate that FGF can act by transcriptional regulation of a chromatin-modifying gene.

Previous work in differentiating mouse ES cells identified a neural progenitor transition state prior to the onset of neuronal differentiation, which shares some features with cells in the preneural tube that then become neurogenic neural progenitors in the CNT (Abranches et al., 2009). This study generated anterior neural tissue rather than spinal cord and cells underwent more heterogeneous differentiation than *in vivo*. However, it will be interesting to determine the extent to which our findings can be used to manipulate neural differentiation cell states *in vitro*, including later derived populations, such as neuromesodermal progenitors (Tsakiridis et al., 2014). The crucial next steps are to discover how newly associated signals and cellular processes contribute to the mechanism of differentiation and, in particular, how signalling and cell cycle regulation are integrated to promote coordinated epigenetic changes that re-configure chromatin for neural differentiation.

## MATERIALS AND METHODS

### Tissue dissection

Tissues defined in Fig. 1 were dissected for microarray or Helicos Bio Direct RNA Sequencing (DRS) from 10- to 12-somite embryos (HH stage 10; Hamburger and Hamilton, 1951). Dissections were carried out in L15 medium at 4°C and explants pooled in TRIzol reagent (Gibco) for RNA extraction. Notochord was removed by controlled trypsin digestion that aimed to keep the neural ventral midline. For the microarray, at least five tissue samples for each region were pooled to make each of three biological replicates for each (*n*>15 for each region).

### RNA extraction, quality testing and cDNA synthesis

RNA extraction, quality testing and cDNA synthesis were carried out using standard procedures (see methods in the supplementary material).

### Cloning and whole-mount mRNA *in situ* hybridisation

Plasmids or analysis of mRNA expression were generated by PCR and cloning, were ESTs, or were kindly provided by colleagues (see methods in the supplementary material). Standard methods for whole-mount *in situ* hybridisation were used to detect mRNA expression.

### Bead-grafting experiments

Heparin-coated beads were soaked in FGF4 or FGF8b, or washed in PBS and grafted in contact with the stem zone epiblast in 10-somite embryos established in EC culture as described previously (Diez del Corral et al., 2002).

### Microarray analysis

Microarray data were normalised and expression measures computed using the Robust Multiarray Average (RMA) method (Irizarry et al., 2003). Hierarchical clustering confirmed that the biological replicates accurately reflected distinct groups. Simple inter-replicate expression plots, where probe intensities for two replicates within each condition are plotted against each other, were used to ensure that no large-scale systematic effects are present in the data. A linear model was fit to the normalised intensity data using the R package ‘limma’ (Smyth, 2004; Smyth et al., 2005). *P*-values for the resulting fold changes are calculated by shrinkage of the empirical Bayes-moderated t-statistic for each probe and are then adjusted for multiple hypothesis testing by controlling the False Discovery Rate (FDR) (using the correction detailed by Benjamini and Hochberg, 1995) to produce q-values for linear model analysis). Probe-sets with q<0.046 were considered significantly regulated. Annotations for the microarray probe-sets were obtained from the R annotation database ‘chicken.db’ along with additional novel annotation strategies (see methods in the supplementary material).

### Strategies for additional chicken gene annotation

If there was no additional information in the latest annotation from Affymetrix, we identified those probe-sets with known EST/cDNA identifiers and associated these with predicted Ensembl/Entrez genes predicted from the genome or if not present in the genome assembly, associated with orthologs from other species. Not all EST/cDNA identifiers mapped to a predicted gene and may represent novel coding or non-coding genes. The Ensembl Compara database was used to map these genes to human orthologs where necessary for functional annotation purposes. All 1:1 chicken:human orthologs were assumed to be true orthologs and, in addition, conservation of gene order between these two species was used to resolve orthologues for 1:many and many:many relationships. A further strategy used PsiBLAST (Altschul et al., 1997) to identify known proteins in the UniProt database (The Uniprot Consortium, 2010) with high sequence identity to the translated oligo consensus DNA sequence for poorly annotated probe-sets. These processes resulted in gene associations for 1678 poorly annotated probe-sets focussed on the poorly annotated probe-sets in the lists of significantly regulated genes for each tissue comparison. The updated annotations have a considerable impact on the annotation coverage for these sets of genes, with the un-annotated fraction dropping from ∼35% to ∼16% for all the tissue comparisons.

### Systems biology pathway analysis

Hypergeometric tests, computed via the R library ‘GOstats’, were applied to the lists of significantly regulated probe-sets for a given tissue comparison. This allowed the identification of GO terms and KEGG pathways that were significantly enriched within the set of significant genes. The hypergeometric test assigns a *P*-value for each GO term and pathway based on the probability of having the same (or greater) number of probe-sets with that GO/KEGG annotation in a randomly drawn sample that is the same size as the set of significant genes.

### Cell cycle analysis

To calculate cell cycle and S phase duration, HH10 embryos were exposed to EdU for defined periods (1 or 4 h), fixed and processed to detect EdU-labelled DNA using Click-iT EdU kit (Molecular Probes) following manufacturer's instructions. The percentage of EdU-positive cells was determined by counting EdU+ cells/all nuclei (DAPI labelled) in five sections through each region in five embryos for each exposure time. Values obtained were then used in the following simultaneous equations, after (Storey, 1989):
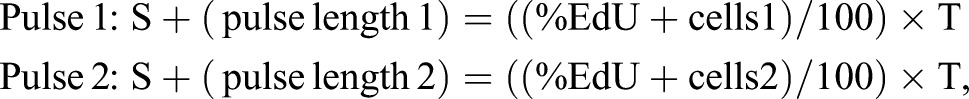
where T=total cell-cycle time and S=S phase length. T was calculated by subtracting equations:
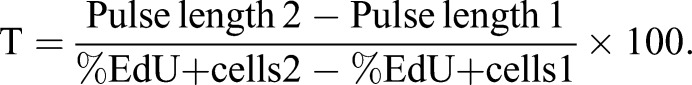


S phase length was determined by substituting the calculated value for T into one of the original equations. Mitotic index was determined by counting phospho-H3-positive cells/all nuclei (DAPI labelled) in five sections through each region in five embryos for each exposure time. Statistical comparisons were made using Student's *t*-test (Fig. 5B).

### Aggresome assay

Aggresomes were detected using ProteoStat Aggresome Detection Kit (Enzo; ENZ-51035-K100) following manufacturer's instructions. Fluorescence intensities were measured using the ROI manager in FIJI on maximum projections and normalised to background levels (Fig. 5E).

### Data access

The raw microarray data have been deposited in the ArrayExpress public repository at the EBI (E-MTAB-2734) in MIAME-compliant format (and see supplementary material Table S1), and the raw DRS data have been deposited in the ENA public repository (accession number PRJEB6699). In addition, processed datasets for the DRS data are available from https://www.compbio.dundee.ac.uk/polyADB/ in a variety of formats.

## Supplementary Material

Supplementary Material
