# Supplementary material for: Major transcriptome re-organisation and abrupt changes in signalling, cell cycle and chromatin regulation at neural differentiation in vivo
Source: Development. 2014 Aug;141(16):3266–76. doi: 10.1242/dev.112623 (PMC4197544; doi:10.1242/dev.112623)
Supplement: Supplementary Material [file supp_141_16_3266__index.html]

Major transcriptome re-organisation and abrupt changes in signalling, cell cycle and chromatin regulation at neural differentiation in vivo — Supplementary Material 

# Major transcriptome re-organisation and abrupt changes in signalling, cell cycle and chromatin regulation at neural differentiation *in vivo*

## DEV112623 Supplementary Material

**Files in this Data Supplement:**

- **Supplementary Material**
