# Supplementary material for: Major transcriptome re-organisation and abrupt changes in signalling, cell cycle and chromatin regulation at neural differentiation in vivo
Source: Development. 2014 Aug;141(16):3266–76. doi: 10.1242/dev.112623 (PMC4197544; doi:10.1242/dev.112623)
Supplement: Supplementary Material [file supp_141.16.3266_DEV112623.pdf]

Supplementary Methods

RNA extraction, quality testing and cDNA synthesis

All surfaces and dissecting tools were treated with RNAZap (Ambion) and rinsed with DEPC-treated water. RNA was extracted in Trizol reagent (Invitrogen) by phase separation with chloroform, followed by precipitation with isopropanol and linear acrylamide. The RNA was washed in 70% ethanol, air-dried, resuspended in DEPC-water and frozen in liquid nitrogen. Total RNA was quantified and quality tested using the Agilent RNA assay (Agilent Bioanalyser pico RNA chip) by ARK-Genomics. Samples with a RIN number above 8.0 where selected, RNA was reverse transcribed, amplified x2 labelled and hybridized to the Affymetrix GeneChip Chicken Genome Array.

Cloning and wholemount mRNA in situ hybridisation

PCR amplification of desired gene sequences (primer sequences available on request) from HH22 cDNA and were cloned into pSCA/B (Strataclone) (*EP300, FABP7, FDFT1, GREB1, HDAC1, HMGCR3, IDI, JARID2, LSS, Mid1, NCAM, PSMA7, PSMB1, PSMD3, PSMD7, PSME3, SMARCA2, WEE1*), purchased from ARK Genomics for antisense mRNA synthesis (*AXIN2* ChEST 755616; *BTG2* ChEST 835n23; *FABP5* ChEST 75n20; *NCAM* ChEST 463a15; *Ret* ChEST924e6; or kindly provided, *CYCLIN-D1*, A. Munsterberg, *CYCLIN-E1*, *FGF8,* G. Martin, *FGFR2*, E. Pasquale, *FZD1*, P. Francis-West, *HES5.1* D. Henrique, *HOXB4*, R. Krumlauf, *IGFBP2*, G. Allen, *ID2*, M. Bronner, *PTC1* C. Tabin, *PAX6*, J. Briscoe, *RARb*, D. Dhouailly, *SMAD6*, C. Stern, *WNT8c*, J. Dodd, *SPRY2*, G.Martin).

Control for potential sex bias The chick embryo exhibits early sex specific differences in gene expression (Zhang et al., 2010; Zhao et al., 2010). These are largely due to lack of dosage compensation for genes on the Z chromosome and a smaller number of W genes (males are ZZ, females ZW). Differential expression of sex identity genes is likely to be masked by sample pooling, but to assess any potential bias significantly regulated gene lists were compared with a list of Cell Autonomous Sex Identity (CASI) genes (M. Clinton and colleagues (Zhao et al., 2010) and pers. comm.). This indicated no consistent up regulation of CASI genes, for example, only 6 of 259 CASI genes are upregulated in PNT in comparison with CNT, and none of these genes were included in significantly regulated pathways identified by KEGG analysis.

Comparison with Direct RNA sequencing (DRS) data

Direct RNA sequencing was carried out on three biological replicates of PNT tissue from individual HH10 embryos. Total RNA was extracted from the biological replicates using standard procedures (as above) and was then sequenced by Helicos Biosciences, producing between 7.2 to 16.4 million reads per sample. The raw reads were mapped to v2.1 of the chicken genome (galGal3) with Helicos Bio's proprietary mapping pipeline (v2.0.022410) with the default parameters. The mapped reads were then filtered with four additional selection criteria to remove as much noise from the data as possible (details available on request). The DRS PNT data finally of a total of 5,178 ensembl genes with measured expression in all three PNT.

Probability evaluation for conserved gene cohort We assessed the chance that the 29 conserved RNA processing and cell cycle regulating genes represent an entirely random result by simulating $1x10^5$ random draws of 80 genes from the population of genes sampled by the chicken microarray. We then examined the number of genes from these random draws that showed a statistically significant ($>2\sigma$) fold-change in any of the tissue comparisons. Of the $1x10^5$ random draws, 104 contained 29 or more genes with a significant fold change, indicating that the probability of a random set of 80 genes containing the same number of regulated genes that we observe in the orthologous set is small ($p = 0.001$).

References

1. Altschul SF*, et al.* (1997) Gapped BLAST and PSI-BLAST: a new generation of protein database search programs. (Translated from eng) *Nucleic Acids Res* 25(17):3389-3402 (in eng).

* see Geisha website: http://geisha.arizona.edu/geisha/search

References

Adams MS, Gammill LS, Bronner-Fraser M. 2008. Discovery of transcription factors and other candidate regulators of neural crest development. *Developmental dynamics : an official publication of the American Association of Anatomists* **237**: 1021-1033.

Aglyamova GV, Agarwala S. 2007. Gene expression analysis of the hedgehog signaling cascade in the chick midbrain and spinal cord. *Developmental dynamics : an official publication of the American Association of Anatomists* **236**: 1363-1373.

Akai J, Storey K. 2002. Expression of gamma-adducin is associated with regions of morphogenetic cell movement in the chick embryo. *Mechanisms of development* **119 Suppl 1**: S191-195.

Baker RK, Antin PB. 2003. Ephs and ephrins during early stages of chick embryogenesis. *Developmental dynamics : an official publication of the American Association of Anatomists* **228**: 128-142.

Bao ZZ, Jin Z. 2006. Sema3D and Sema7A have distinct expression patterns in chick embryonic development. *Developmental dynamics : an official publication of the American Association of Anatomists* **235**: 2282-2289.

Basch ML, Bronner-Fraser M, Garcia-Castro MI. 2006. Specification of the neural crest occurs during gastrulation and requires Pax7. *Nature* **441**: 218-222.

Bel-Vialar S, Itasaki N, Krumlauf R. 2002. Initiating Hox gene expression: in the early chick neural tube differential sensitivity to FGF and RA signaling subdivides the HoxB genes in two distinct groups. *Development* **129**: 5103-5115.

Bell KM, Western PS, Sinclair AH. 2000. SOX8 expression during chick embryogenesis. *Mechanisms of development* **94**: 257-260.

Bothe I, Dietrich S. 2006. The molecular setup of the avian head mesoderm and its implication for craniofacial myogenesis. *Developmental dynamics : an official publication of the American Association of Anatomists* **235**: 2845-2860.

Chambers D, Mason I. 2000. Expression of sprouty2 during early development of the chick embryo is coincident with known sites of FGF signalling. *Mechanisms of development* **91**: 361-364.

Chapman SC, Brown R, Lees L, Schoenwolf GC, Lumsden A. 2004. Expression analysis of chick Wnt and frizzled genes and selected inhibitors in early chick patterning. *Developmental dynamics : an official publication of the American Association of Anatomists* **229**: 668-676.

Cheng Y, Cheung M, Abu-Elmagd MM, Orme A, Scotting PJ. 2000. Chick sox10, a transcription factor expressed in both early neural crest cells and central nervous system. *Brain Res Dev Brain Res* **121**: 233-241.

Cooper O, Sweetman D, Wagstaff L, Munsterberg A. 2008. Expression of avian prickle genes during early development and organogenesis. *Developmental dynamics : an official publication of the American Association of Anatomists* **237**: 1442-1448.

Crossley PH, Minowada G, MacArthur CA, Martin GR. 1996. Roles for FGF8 in the induction, initiation, and maintenance of chick limb development. *Cell* **84**: 127-136.

Dessimoz J, Opoka R, Kordich JJ, Grapin-Botton A, Wells JM. 2006. FGF signaling is necessary for establishing gut tube domains along the anterior-posterior axis in vivo. *Mechanisms of development* **123**: 42-55.
Diez del Corral R, Olivera-Martinez I, Goriely A, Gale E, Maden M, Storey K. 2003. Opposing FGF and retinoid pathways control ventral neural pattern, neuronal differentiation, and segmentation during body axis extension. *Neuron* **40**: 65-79.
Eblaghie MC, Lunn JS, Dickinson RJ, Munsterberg AE, Sanz-Ezquerro JJ, Farrell ER, Mathers J, Keyse SM, Storey K, Tickle C. 2003. Negative feedback regulation of FGF signaling levels by Pyst1/MKP3 in chick embryos. *Current biology : CB* **13**: 1009-1018.
Falls DL, Rosen KM, Corfas G, Lane WS, Fischbach GD. 1993. ARIA, a protein that stimulates acetylcholine receptor synthesis, is a member of the neu ligand family. *Cell* **72**: 801-815.
Fan MJ, Sokol SY. 1997. A role for Siamois in Spemann organizer formation. *Development* **124**: 2581-2589.
Figdor MC, Stern CD. 1993. Segmental organization of embryonic diencephalon. *Nature* **363**: 630-634.
Fior R, Henrique D. 2005. A novel hes5/hes6 circuitry of negative regulation controls Notch activity during neurogenesis. *Dev Biol* **281**: 318-333.
Frolova EI, Fokina VM, Beebe DC. 2004. The expression pattern of opticin during chicken embryogenesis. *Gene expression patterns : GEP* **4**: 335-338.
Gammill LS, Bronner-Fraser M. 2002. Genomic analysis of neural crest induction. *Development* **129**: 5731-5741.
Garcia-Castro MI, Marcelle C, Bronner-Fraser M. 2002. Ectodermal Wnt function as a neural crest inducer. *Science* **297**: 848-851.
Gaunt SJ, Strachan L. 1996. Temporal colinearity in expression of anterior Hox genes in developing chick embryos. *Developmental Dynamics* **207**: 270-280.
Ghatpande S, Brand T, Zile M, Evans T. 2006. Bmp2 and Gata4 function additively to rescue heart tube development in the absence of retinoids. *Developmental dynamics : an official publication of the American Association of Anatomists* **235**: 2030-2039.
Henrique D, Adam J, Myat A, Chitnis A, Lewis J, Ish-Horowicz D. 1995. Expression of a Delta homologue in prospective neurons in the chick. *Nature* **375**: 787-790.
Hume CR, Dodd J. 1993. Cwnt-8C: A novel Wnt gene with a potential role in primitive streak formation and hindbrain organization. *Development* **119**: 1147-1160.
Jin EJ, Erickson CA, Takada S, Burrus LW. 2001. Wnt and BMP signaling govern lineage segregation of melanocytes in the avian embryo. *Dev Biol* **233**: 22-37.
Kee Y, Bronner-Fraser M. 2001a. Id4 expression and its relationship to other Id genes during avian embryonic development. *Mechanisms of development* **109**: 341-345.
-. 2001b. The transcriptional regulator Id3 is expressed in cranial sensory placodes during early avian embryonic development. *Mechanisms of development* **109**: 337-340.
Khudyakov J, Bronner-Fraser M. 2009. Comprehensive spatiotemporal analysis of early chick neural crest network genes. *Developmental dynamics : an official publication of the American Association of Anatomists* **238**: 716-723.
Kispert A, Ortner H, Cooke J, Herrmann BG. 1995. The chick Brachyury gene: Developmental expression pattern and response to axial induction by localized activin. *Developmental Biology* **168**: 406-415.

Klewer SE, Yatskievych T, Pogreba K, Stevens MV, Antin PB, Camenisch TD. 2006. Has2 expression in heart forming regions is independent of BMP signaling. *Gene expression patterns : GEP* **6**: 462-470.

Knecht AK, Harland RM. 1997. Mechanisms of dorsal-ventral patterning in noggin-induced neural tissue. *Development* **124**: 2477-2488.

Kos R, Reedy MV, Johnson RL, Erickson CA. 2001. The winged-helix transcription factor FoxD3 is important for establishing the neural crest lineage and repressing melanogenesis in avian embryos. *Development* **128**: 1467-1479.

LeClerc C, Daguzan C, Nicolas MT, Chabret C, Duprat AM, Moreau M. 1997. L-type calcium channel activation controls the in vivo transduction of the neuralizing signal in the amphibian embryos. *Mechanisms of development* **64**: 105-110.

Liem KF, Jr., Tremml G, Jessell TM. 1997. A role for the roof plate and its resident TGFbeta-related proteins in neuronal patterning in the dorsal spinal cord. *Cell* **91**: 127-138.

Lobjois V, Benazeraf B, Bertrand N, Medevielle F, Pituello F. 2004. Specific regulation of cyclins D1 and D2 by FGF and Shh signaling coordinates cell cycle progression, patterning, and differentiation during early steps of spinal cord development. *Dev Biol* **273**: 195-209.

Lunn JS, Fishwick KJ, Halley PA, Storey KG. 2007. A spatial and temporal map of FGF/Erk1/2 activity and response repertoires in the early chick embryo. *Dev Biol* **302**: 536-552.

Maden M. 1994. Distribution of cellular retinoic acid-binding proteins I and II in the chick embryo and their relationship to teratogenesis. *Teratology* **50**: 294-301.

Marom K, Shapira E, Fainsod A. 1997. The chicken caudal genes establish an anterior-posterior gradient by partially overlapping temporal and spatial patterns of expression. *Mechanisms of development* **64**: 41-52.

McCabe KL, Shiau CE, Bronner-Fraser M. 2007. Identification of candidate secreted factors involved in trigeminal placode induction. *Developmental dynamics : an official publication of the American Association of Anatomists* **236**: 2925-2935.

McKeown SJ, Lee VM, Bronner-Fraser M, Newgreen DF, Farlie PG. 2005. Sox10 overexpression induces neural crest-like cells from all dorsoventral levels of the neural tube but inhibits differentiation. *Developmental dynamics : an official publication of the American Association of Anatomists* **233**: 430-444.

Monaghan AP, Kioschis P, Wu W, Zuniga A, Bock D, Poustka A, Delius H, Niehrs C. 1999. Dickkopf genes are co-ordinately expressed in mesodermal lineages. *Mechanisms of development* **87**: 45-56.

Morgan R, Sargent MG. 1997. The role in neural patterning of translation initiation factor eIF4AII; induction of neural fold genes. *Development* **124**: 2751-2760.

Myat A, Henrique D, Ish Horowicz D, Lewis J. 1996. A chick homologue of Serrate and its relationship with Notch and Delta homologues during central neurogenesis. *Developmental Biology* **174**: 233-247.

Niss K, Leutz A. 1998. Expression of the homeobox gene GBX2 during chicken development. *Mechanisms of development* **76**: 151-155.

Ogura K, Matsumoto K, Kuroiwa A, Isobe T, Otoguro T, Jurecic V, Baldini A, Matsuda Y, Ogura T. 2001. Cloning and chromosome mapping of human and chicken Iroquois (IRX) genes. *Cytogenet Cell Genet* **92**: 320-325.

Ohuchi H, Kimura S, Watamoto M, Itoh N. 2000. Involvement of fibroblast growth factor (FGF)18-FGF8 signaling in specification of left-right asymmetry and brain and limb development of the chick embryo. *Mechanisms of development* **95**: 55-66.

Palmeirim I, Henrique D, Ish Horowicz D, Pourquie O. 1997. Avian hairy gene expression identifies a molecular clock linked to vertebrate segmentation and somitogenesis. *Cell* **91**: 639-648.

Paxton CN, Bleyl SB, Chapman SC, Schoenwolf GC. 2010. Identification of differentially expressed genes in early inner ear development. *Gene expression patterns : GEP* **10**: 31-43.

Pituello F, Medevielle F, Foulquier F, Duprat AM. 1999. Activation of Pax6 depends on somitogenesis in the chick embryo cervical spinal cord. *Development* **126**: 587-596.

Qiu M, Shimamura K, Sussel L, Chen S, Rubenstein JL. 1998. Control of anteroposterior and dorsoventral domains of Nkx-6.1 gene expression relative to other Nkx genes during vertebrate CNS development. *Mechanisms of development* **72**: 77-88.

Rangini Z, Ben-Yehuda A, Shapira E, Gruenbaum Y, Fainsod A. 1991. CHox E, a chicken homeogene of the H2.0 type exhibits dorso-ventral restriction in the proliferating region of the spinal cord. *Mechanisms of development* **35**: 13-24.

Rodrigues S, Santos J, Palmeirim I. 2006. Molecular characterization of the rostral-most somites in early somitic stages of the chick embryo. *Gene expression patterns : GEP* **6**: 673-677.

Stein S, Kessel M. 1995. A homeobox gene involved in node, notochord and neural plate formation of chick embryos. *Mechanisms of development* **49**: 37-48.

Storm R, Cholewa-Waclaw J, Reuter K, Brohl D, Sieber M, Treier M, Muller T, Birchmeier C. 2009. The bHLH transcription factor Olig3 marks the dorsal neuroepithelium of the hindbrain and is essential for the development of brainstem nuclei. *Development* **136**: 295-305.

Streit A, Thery C, Stern CD. 1994. Of mice and frogs. *Trends in Genetics* **10**: 181-183.

Swindell EC, Thaller C, Sockanathan S, Petkovich M, Jessell TM, Eichele G. 1999. Complementary domains of retinoic acid production and degradation in the early chick embryo. *Dev Biol* **216**: 282-296.

Terry K, Magan H, Baranski M, Burrus LW. 2000. Sfrp-1 and sfrp-2 are expressed in overlapping and distinct domains during chick development. *Mechanisms of development* **97**: 177-182.

Toyoda R, Nakamura H, Watanabe Y. 2005. Identification of protogenin, a novel immunoglobulin superfamily gene expressed during early chick embryogenesis. *Gene expression patterns : GEP* **5**: 778-785.

Uchikawa M, Yoshida M, Iwafuchi-Doi M, Matsuda K, Ishida Y, Takemoto T, Kondoh H. 2011. B1 and B2 Sox gene expression during neural plate development in chicken and mouse embryos: universal versus species-dependent features. *Development, growth & differentiation* **53**: 761-771.

Uwanogho D, Rex M, Cartwright EJ, Pearl G, Healy C, Scotting PJ, Sharpe PT. 1995. Embryonic expression of the chicken Sox2, Sox3 and Sox11 genes suggests an interactive role in neuronal development. *Mechanisms of development* **49**: 23-36.

Walshe J, Mason I. 2000. Expression of FGFR1, FGFR2 and FGFR3 during early neural development in the chick embryo. *Mechanisms of development* **90**: 103-110.

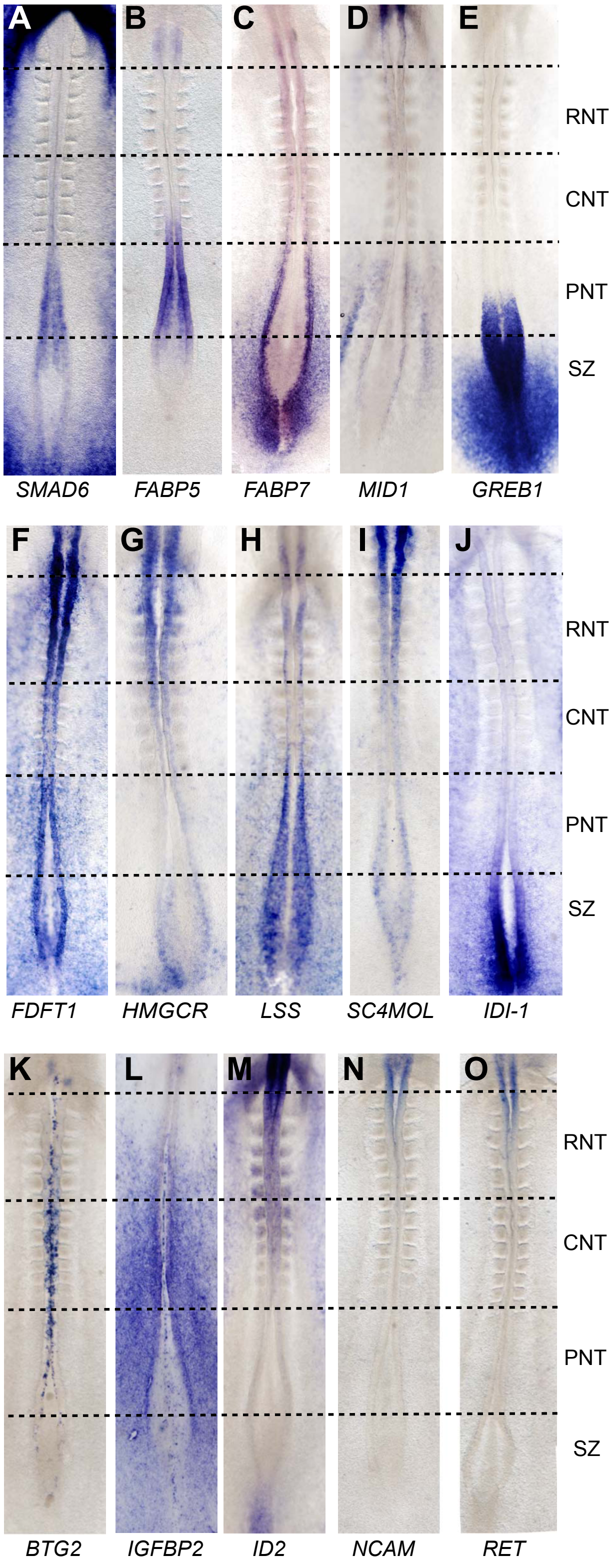

Olivera-Martinez et al. Figure S1

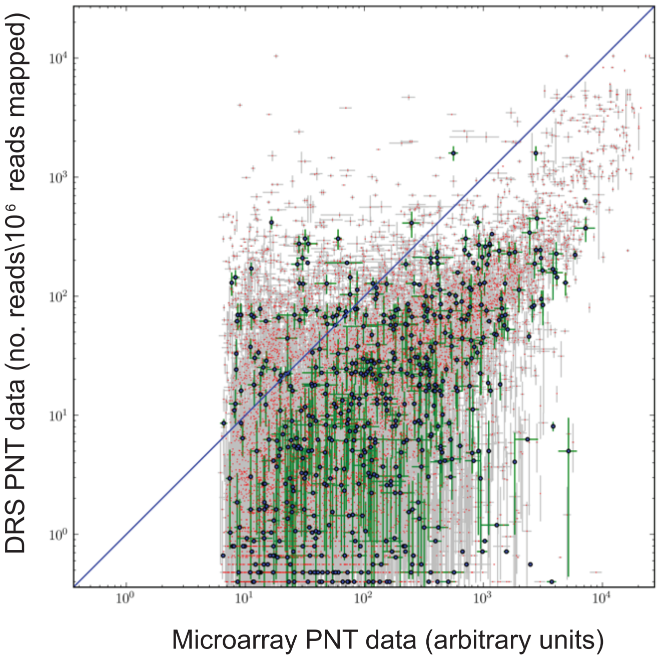

Olivera-Martinez et al. Figure S2

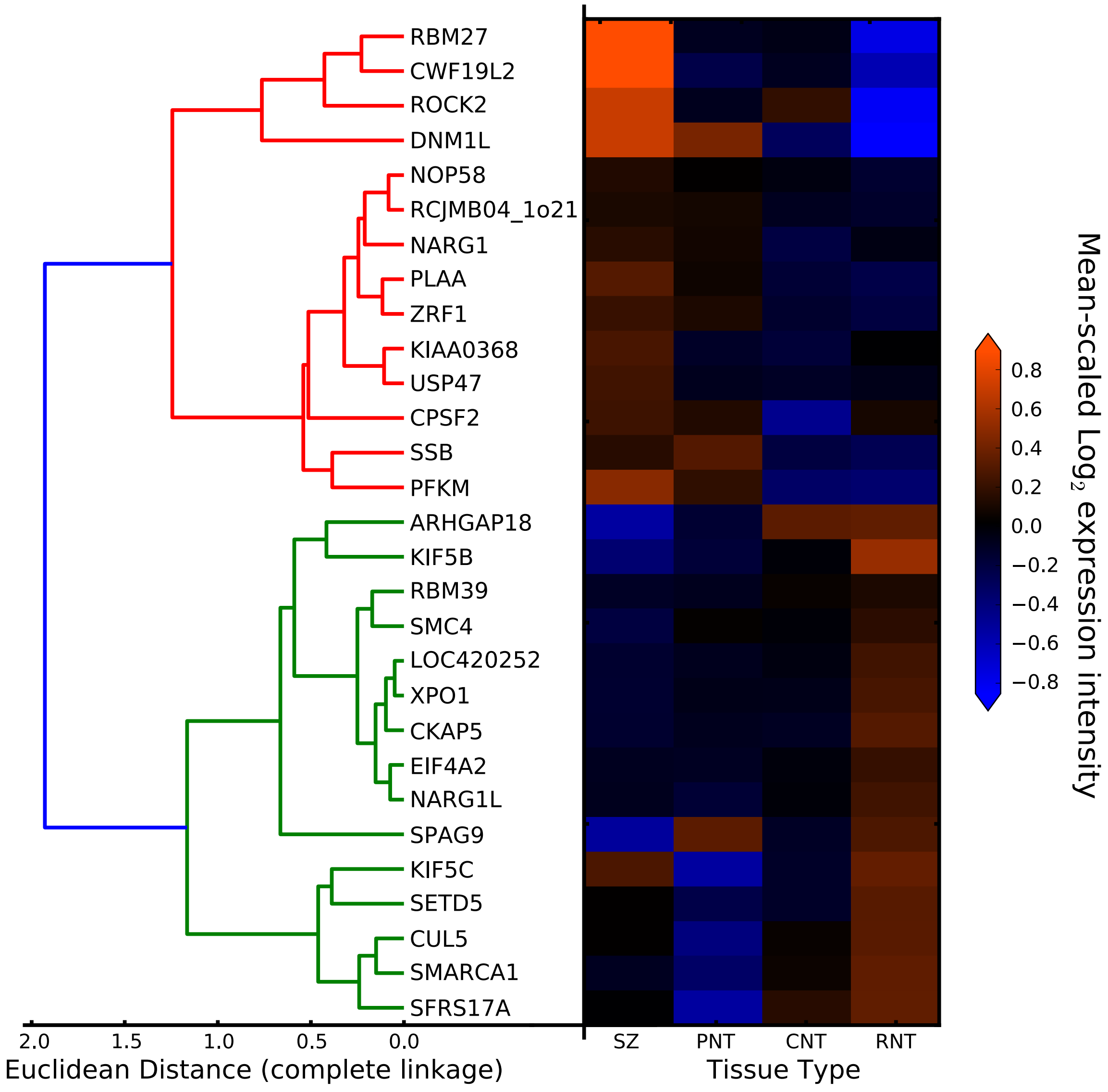

Olivera-Martinez et al Figure S3

Supplementary figure legends

Figure S1 Expression patterns of genes not previously shown to be differentially expressed along the elongating neural axis
Fifteen genes with restricted expression patterns along the neural axis indicated by microarray analysis were cloned and labelled mRNA probes made for whole embryo in situ hybridisation to reveal expression patterns at HH stage 10-11. A-J genes expressed higher in the preneural tube (PNT) than caudal neural tube (CNT), including 6 genes associated with steroid biogenesis/signalling, Greb1, FDFT1, HMGCR3, LSS and SC4MOL, IDI-1; K, L genes expressed higher in the caudal neural tube than preneural tube; M-O, genes higher in the RNT than in the caudal neural tube. Ten further predicted expression patterns for cell cycle and proteasome genes are presented in Figure 5.

Figure S2 Comparison of microarray and DRS data for the Preneural Tube
Plot of 5178 Ensembl genes with measured expression in both the DRS and microarray datasets, showing a good correlation (Pearson correlation coefficient $r = 0.68$, where 1 is a perfect correlation, -1 is a perfect anti-correlation and 0 corresponds to un-correlated datasets). High-lighted in blue are the 353 genes within this 5,178 that were found to be significantly ($>2\sigma$) differentially expressed between the PNT and CNT tissues in the microarray analysis. These genes are distributed over the full extent of the graph, demonstrating that the differential expression analysis results from the microarray data are not strongly affected by sampling effects, and the expression levels for these genes results are broadly robust to the technology used to measure their transcription.

Figure S3 Conservation of downregulated RNA processing and cell cycle genes at neural differentiation onset Expression heatmap of the 29 genes found to be orthologous to the 65 *Drosophila* homologues of mouse cluster V genes identified by (Mitiku and Baker 2007). The heatmap shows the log-base 2 of the absolute expression intensities for the genes in each of the four tissues, scaled so that the mean value of expression from summing all four tissue intensities is the same for each gene. The hierarchical clustering was computed with the most conservative (complete) linkage. The genes cluster into two broad groups, but most indicate reduced expression as differentiation commences (see text).

Table S1.

Download Table S1

Table S2 Genes known to be expressed in restricted domains along the neural axis were represented as expected in the microarray

Comparison	Exemplar genes
RNT > CNT	*Sox10* (Cheng et al. 2000), *NRG-1 (Falls et al. 1993), SFRP-1(Terry et al. 2000), FZD1 (Fz1) (Chapman et al. 2004), Zic1(Khudyakov and Bronner-Fraser 2009), Sema3D (Bao and Jin 2006).*
CNT > RNT	*Cdx2 (Marom et al. 1997), Cdx4 (Marom et al. 1997), Ahcyl1 (Gammill and Bronner-Fraser 2002), Hoxb7 (Bel-Vialar et al. 2002), Hoxb4 (Gaunt and Strachan 1996)*
CNT > PNT	*Ptch1 (Aglyamova and Agarwala 2007), Pax6 (Pituello et al. 1999), Pax7 (Basch et al. 2006), Pax3 (Bothe and Dietrich 2006) Irx1 (Ogura et al. 2001), Irx3 (Diez del Corral et al. 2003), Dbx2 (Rangini et al. 1991), Dkk3 (Monaghan et al. 1999) Foxd3 (Kos et al. 2001), Sox9 (McKeown et al. 2005), Cdx1 (Marom et al. 1997), Id1 (Kee and Bronner-Fraser 2001a), Id3 (Kee and Bronner-Fraser 2001b), Bmp7 (Liem et al. 1997), Hes1 (Palmeirim et al. 1997), Hes5 (Fior and Henrique 2005), Hes6 (Fior and Henrique 2005), Jag1 (Serrate1) (Myat et al. 1996), Lfng (Rodrigues et al. 2006), RARβ (Diez del Corral et al. 2003), Sox8 (Bell et al. 2000), Fgfr3 (Walshe and Mason 2000), Nkx6.2 (Diez del Corral et al. 2003), Nkx6.1 (Qiu et al. 1998), Crabp1 (Maden 1994), Snail2*, FoxD3 (Adams et al. 2008), Notch1*,Optc (Frolova et al. 2004), Wnt4 .*
PNT > CNT	*Spry1 (Dessimoz et al. 2006), Spry2 (Chambers and Mason 2000), Dusp6 (Eblaghie et al. 2003), Add3 (Akai and Storey 2002), Prickle1 (Cooper et al. 2008), Wnt8C (Hume and Dodd 1993), Fgf18 (Ohuchi et al. 2000), CyclinD2 (Lobjois et al. 2004), Sox2 (Uchikawa et al. 2011), Nolc (Gammill and Bronner-Fraser 2002), Etv5 (Lunn et al. 2007).*
PNT > SZ	*Sox3 (Uwanogho et al. 1995), NeuroG2 (Diez del Corral et al. 2003), Gbx2 (Niss and Leutz 1998), Olig3 (Storm et al. 2009), Fgfr2 (Lunn et al. 2007), Pdlim4*, Prtg (Toyoda et al. 2005), Sfrp2*.*
SZ > PNT	*Bra (Kispert et al. 1995), Delta1 (Henrique et al. 1995), Cyp26a (Swindell et al. 1999), cNot-1 (Stein and Kessel 1995), Bmp2 (Ghatpande et al. 2006), EphA4 (Baker and Antin 2003), Wnt3a (Jin et al. 2001). Fgf8 (Crossley et al. 1996), Fgf3 (Paxton et al. 2010), Fgf19*, Msx1(Khudyakov and Bronner-Fraser 2009), cMyc (Khudyakov and Bronner-Fraser 2009), Wnt5a (Garcia-Castro et al. 2002), Wnt5b (Garcia-Castro et al. 2002), Akap12*, Epha1*, Epha4 (Bothe and Dietrich 2006), Etv1 (Lunn et al. 2007), Fzd10 (McCabe et al. 2007), Has2 (Klewer et al. 2006).*

* see Geisha website: http://geisha.arizona.edu/geisha/search

References

Adams MS, Gammill LS, Bronner-Fraser M. 2008. Discovery of transcription factors and other candidate regulators of neural crest development. *Developmental dynamics : an official publication of the American Association of Anatomists* **237**: 1021-1033.

Aglyamova GV, Agarwala S. 2007. Gene expression analysis of the hedgehog signaling cascade in the chick midbrain and spinal cord. *Developmental dynamics : an official publication of the American Association of Anatomists* **236**: 1363-1373.

Akai J, Storey K. 2002. Expression of gamma-adducin is associated with regions of morphogenetic cell movement in the chick embryo. *Mechanisms of development* **119 Suppl 1**: S191-195.

Baker RK, Antin PB. 2003. Ephs and ephrins during early stages of chick embryogenesis. *Developmental dynamics : an official publication of the American Association of Anatomists* **228**: 128-142.

Bao ZZ, Jin Z. 2006. Sema3D and Sema7A have distinct expression patterns in chick embryonic development. *Developmental dynamics : an official publication of the American Association of Anatomists* **235**: 2282-2289.

Basch ML, Bronner-Fraser M, Garcia-Castro MI. 2006. Specification of the neural crest occurs during gastrulation and requires Pax7. *Nature* **441**: 218-222.

Bel-Vialar S, Itasaki N, Krumlauf R. 2002. Initiating Hox gene expression: in the early chick neural tube differential sensitivity to FGF and RA signaling subdivides the HoxB genes in two distinct groups. *Development* **129**: 5103-5115.

Bell KM, Western PS, Sinclair AH. 2000. SOX8 expression during chick embryogenesis. *Mechanisms of development* **94**: 257-260.

Bothe I, Dietrich S. 2006. The molecular setup of the avian head mesoderm and its implication for craniofacial myogenesis. *Developmental dynamics : an official publication of the American Association of Anatomists* **235**: 2845-2860.

Chambers D, Mason I. 2000. Expression of sprouty2 during early development of the chick embryo is coincident with known sites of FGF signalling. *Mechanisms of development* **91**: 361-364.

Chapman SC, Brown R, Lees L, Schoenwolf GC, Lumsden A. 2004. Expression analysis of chick Wnt and frizzled genes and selected inhibitors in early chick patterning. *Developmental dynamics : an official publication of the American Association of Anatomists* **229**: 668-676.

Cheng Y, Cheung M, Abu-Elmagd MM, Orme A, Scotting PJ. 2000. Chick sox10, a transcription factor expressed in both early neural crest cells and central nervous system. *Brain Res Dev Brain Res* **121**: 233-241.

Cooper O, Sweetman D, Wagstaff L, Munsterberg A. 2008. Expression of avian prickle genes during early development and organogenesis. *Developmental dynamics : an official publication of the American Association of Anatomists* **237**: 1442-1448.

Crossley PH, Minowada G, MacArthur CA, Martin GR. 1996. Roles for FGF8 in the induction, initiation, and maintenance of chick limb development. *Cell* **84**: 127-136.

Dessimoz J, Opoka R, Kordich JJ, Grapin-Botton A, Wells JM. 2006. FGF signaling is necessary for establishing gut tube domains along the anterior-posterior axis in vivo. *Mechanisms of development* **123**: 42-55.
Diez del Corral R, Olivera-Martinez I, Goriely A, Gale E, Maden M, Storey K. 2003. Opposing FGF and retinoid pathways control ventral neural pattern, neuronal differentiation, and segmentation during body axis extension. *Neuron* **40**: 65-79.
Eblaghie MC, Lunn JS, Dickinson RJ, Munsterberg AE, Sanz-Ezquerro JJ, Farrell ER, Mathers J, Keyse SM, Storey K, Tickle C. 2003. Negative feedback regulation of FGF signaling levels by Pyst1/MKP3 in chick embryos. *Current biology : CB* **13**: 1009-1018.
Falls DL, Rosen KM, Corfas G, Lane WS, Fischbach GD. 1993. ARIA, a protein that stimulates acetylcholine receptor synthesis, is a member of the neu ligand family. *Cell* **72**: 801-815.
Fan MJ, Sokol SY. 1997. A role for Siamois in Spemann organizer formation. *Development* **124**: 2581-2589.
Figdor MC, Stern CD. 1993. Segmental organization of embryonic diencephalon. *Nature* **363**: 630-634.
Fior R, Henrique D. 2005. A novel hes5/hes6 circuitry of negative regulation controls Notch activity during neurogenesis. *Dev Biol* **281**: 318-333.
Frolova EI, Fokina VM, Beebe DC. 2004. The expression pattern of opticin during chicken embryogenesis. *Gene expression patterns : GEP* **4**: 335-338.
Gammill LS, Bronner-Fraser M. 2002. Genomic analysis of neural crest induction. *Development* **129**: 5731-5741.
Garcia-Castro MI, Marcelle C, Bronner-Fraser M. 2002. Ectodermal Wnt function as a neural crest inducer. *Science* **297**: 848-851.
Gaunt SJ, Strachan L. 1996. Temporal colinearity in expression of anterior Hox genes in developing chick embryos. *Developmental Dynamics* **207**: 270-280.
Ghatpande S, Brand T, Zile M, Evans T. 2006. Bmp2 and Gata4 function additively to rescue heart tube development in the absence of retinoids. *Developmental dynamics : an official publication of the American Association of Anatomists* **235**: 2030-2039.
Henrique D, Adam J, Myat A, Chitnis A, Lewis J, Ish-Horowicz D. 1995. Expression of a Delta homologue in prospective neurons in the chick. *Nature* **375**: 787-790.
Hume CR, Dodd J. 1993. Cwnt-8C: A novel Wnt gene with a potential role in primitive streak formation and hindbrain organization. *Development* **119**: 1147-1160.
Jin EJ, Erickson CA, Takada S, Burrus LW. 2001. Wnt and BMP signaling govern lineage segregation of melanocytes in the avian embryo. *Dev Biol* **233**: 22-37.
Kee Y, Bronner-Fraser M. 2001a. Id4 expression and its relationship to other Id genes during avian embryonic development. *Mechanisms of development* **109**: 341-345.
-. 2001b. The transcriptional regulator Id3 is expressed in cranial sensory placodes during early avian embryonic development. *Mechanisms of development* **109**: 337-340.
Khudyakov J, Bronner-Fraser M. 2009. Comprehensive spatiotemporal analysis of early chick neural crest network genes. *Developmental dynamics : an official publication of the American Association of Anatomists* **238**: 716-723.
Kispert A, Ortner H, Cooke J, Herrmann BG. 1995. The chick Brachyury gene: Developmental expression pattern and response to axial induction by localized activin. *Developmental Biology* **168**: 406-415.

Klewer SE, Yatskievych T, Pogreba K, Stevens MV, Antin PB, Camenisch TD. 2006. Has2 expression in heart forming regions is independent of BMP signaling. *Gene expression patterns : GEP* **6**: 462-470.
Knecht AK, Harland RM. 1997. Mechanisms of dorsal-ventral patterning in noggin-induced neural tissue. *Development* **124**: 2477-2488.
Kos R, Reedy MV, Johnson RL, Erickson CA. 2001. The winged-helix transcription factor FoxD3 is important for establishing the neural crest lineage and repressing melanogenesis in avian embryos. *Development* **128**: 1467-1479.
LeClerc C, Daguzan C, Nicolas MT, Chabret C, Duprat AM, Moreau M. 1997. L-type calcium channel activation controls the in vivo transduction of the neuralizing signal in the amphibian embryos. *Mechanisms of development* **64**: 105-110.
Liem KF, Jr., Tremml G, Jessell TM. 1997. A role for the roof plate and its resident TGFbeta-related proteins in neuronal patterning in the dorsal spinal cord. *Cell* **91**: 127-138.
Lobjois V, Benazeraf B, Bertrand N, Medevielle F, Pituello F. 2004. Specific regulation of cyclins D1 and D2 by FGF and Shh signaling coordinates cell cycle progression, patterning, and differentiation during early steps of spinal cord development. *Dev Biol* **273**: 195-209.
Lunn JS, Fishwick KJ, Halley PA, Storey KG. 2007. A spatial and temporal map of FGF/Erk1/2 activity and response repertoires in the early chick embryo. *Dev Biol* **302**: 536-552.
Maden M. 1994. Distribution of cellular retinoic acid-binding proteins I and II in the chick embryo and their relationship to teratogenesis. *Teratology* **50**: 294-301.
Marom K, Shapira E, Fainsod A. 1997. The chicken caudal genes establish an anterior-posterior gradient by partially overlapping temporal and spatial patterns of expression. *Mechanisms of development* **64**: 41-52.
McCabe KL, Shiau CE, Bronner-Fraser M. 2007. Identification of candidate secreted factors involved in trigeminal placode induction. *Developmental dynamics : an official publication of the American Association of Anatomists* **236**: 2925-2935.
McKeown SJ, Lee VM, Bronner-Fraser M, Newgreen DF, Farlie PG. 2005. Sox10 overexpression induces neural crest-like cells from all dorsoventral levels of the neural tube but inhibits differentiation. *Developmental dynamics : an official publication of the American Association of Anatomists* **233**: 430-444.
Monaghan AP, Kioschis P, Wu W, Zuniga A, Bock D, Poustka A, Delius H, Niehrs C. 1999. Dickkopf genes are co-ordinately expressed in mesodermal lineages. *Mechanisms of development* **87**: 45-56.
Morgan R, Sargent MG. 1997. The role in neural patterning of translation initiation factor eIF4AII; induction of neural fold genes. *Development* **124**: 2751-2760.
Myat A, Henrique D, Ish Horowicz D, Lewis J. 1996. A chick homologue of Serrate and its relationship with Notch and Delta homologues during central neurogenesis. *Developmental Biology* **174**: 233-247.
Niss K, Leutz A. 1998. Expression of the homeobox gene GBX2 during chicken development. *Mechanisms of development* **76**: 151-155.
Ogura K, Matsumoto K, Kuroiwa A, Isobe T, Otoguro T, Jurecic V, Baldini A, Matsuda Y, Ogura T. 2001. Cloning and chromosome mapping of human and chicken Iroquois (IRX) genes. *Cytogenet Cell Genet* **92**: 320-325.
Ohuchi H, Kimura S, Watamoto M, Itoh N. 2000. Involvement of fibroblast growth factor (FGF)18-FGF8 signaling in specification of left-right asymmetry and brain and limb development of the chick embryo. *Mechanisms of development* **95**: 55-66.

Palmeirim I, Henrique D, Ish Horowicz D, Pourquie O. 1997. Avian hairy gene expression identifies a molecular clock linked to vertebrate segmentation and somitogenesis. *Cell* **91**: 639-648.
Paxton CN, Bleyl SB, Chapman SC, Schoenwolf GC. 2010. Identification of differentially expressed genes in early inner ear development. *Gene expression patterns : GEP* **10**: 31-43.
Pituello F, Medevielle F, Foulquier F, Duprat AM. 1999. Activation of Pax6 depends on somitogenesis in the chick embryo cervical spinal cord. *Development* **126**: 587-596.
Qiu M, Shimamura K, Sussel L, Chen S, Rubenstein JL. 1998. Control of anteroposterior and dorsoventral domains of Nkx-6.1 gene expression relative to other Nkx genes during vertebrate CNS development. *Mechanisms of development* **72**: 77-88.
Rangini Z, Ben-Yehuda A, Shapira E, Gruenbaum Y, Fainsod A. 1991. CHox E, a chicken homeogene of the H2.0 type exhibits dorso-ventral restriction in the proliferating region of the spinal cord. *Mechanisms of development* **35**: 13-24.
Rodrigues S, Santos J, Palmeirim I. 2006. Molecular characterization of the rostral-most somites in early somitic stages of the chick embryo. *Gene expression patterns : GEP* **6**: 673-677.
Stein S, Kessel M. 1995. A homeobox gene involved in node, notochord and neural plate formation of chick embryos. *Mechanisms of development* **49**: 37-48.
Storm R, Cholewa-Waclaw J, Reuter K, Brohl D, Sieber M, Treier M, Muller T, Birchmeier C. 2009. The bHLH transcription factor Olig3 marks the dorsal neuroepithelium of the hindbrain and is essential for the development of brainstem nuclei. *Development* **136**: 295-305.
Streit A, Thery C, Stern CD. 1994. Of mice and frogs. *Trends in Genetics* **10**: 181-183.
Swindell EC, Thaller C, Sockanathan S, Petkovich M, Jessell TM, Eichele G. 1999. Complementary domains of retinoic acid production and degradation in the early chick embryo. *Dev Biol* **216**: 282-296.
Terry K, Magan H, Baranski M, Burrus LW. 2000. Sfrp-1 and sfrp-2 are expressed in overlapping and distinct domains during chick development. *Mechanisms of development* **97**: 177-182.
Toyoda R, Nakamura H, Watanabe Y. 2005. Identification of protogenin, a novel immunoglobulin superfamily gene expressed during early chick embryogenesis. *Gene expression patterns : GEP* **5**: 778-785.
Uchikawa M, Yoshida M, Iwafuchi-Doi M, Matsuda K, Ishida Y, Takemoto T, Kondoh H. 2011. B1 and B2 Sox gene expression during neural plate development in chicken and mouse embryos: universal versus species-dependent features. *Development, growth & differentiation* **53**: 761-771.
Uwanogho D, Rex M, Cartwright EJ, Pearl G, Healy C, Scotting PJ, Sharpe PT. 1995. Embryonic expression of the chicken Sox2, Sox3 and Sox11 genes suggests an interactive role in neuronal development. *Mechanisms of development* **49**: 23-36.
Walshe J, Mason I. 2000. Expression of FGFR1, FGFR2 and FGFR3 during early neural development in the chick embryo. *Mechanisms of development* **90**: 103-110.

Table S3 KEGG pathway analysis of Steroid biogenesis genes
Steroid biosynthesis genes upregulated in PNT vs CNT (HSD17B7 and GGPS1 additionally upregulated in SZ+PNT vs CNT+RNT comparison).

Gene name	Chromosome	Log2 FC	p value
HMGCR 3-hydroxy-3-methylglutaryl-CoA reductase	Z	0.87	0.0223
IDI1 isopentenyl-diphosphate delta isomerase 1	2	1.16	0.00159
FDFT1 farnesyl-diphosphate farnesyltransferase 1	3	0.73	0.0002
NSDHL, NAD(P) dependent steroid dehydrogenase-like	4	0.63	0.0188
SC4MOL, C-4 methylsterol oxidase	4	0.97	0.0133
LSS - lanosterol synthase (2,3-oxidosqualene-lanosterol cyclase)	7	0.74	0.0132
HSD17B7 - hydroxysteroid (17-beta) dehydrogenase 7	8	0.53	0.0034
GGPS1 - geranylgeranyl diphosphate synthase 1	3	0.46	0.0022
SREBF2 – sterol regulatory element binding transcription factor 2	1	0.37	0.0218

Table S4 Differential expression of transcription factors in SZ+PNT vs CNT+RNT identified in significantly regulated probeset lists by GO analysis term 0030528

GO term ID	Description	p-value	Genes up and down regulated in SZ +PNT in comparison with CNT+RNT
0030528	Transcription factor activity	0.013	*EGR1, DACH1, SMAD6, HIF1A, CDX2, BETA3, EBF3, NR1H3, HOXA7, SREBF2, HOXA4, CDX4, MSGN1, LOC395448, HOXC-6, MAFK, MAFF, TEAD4, ATF4, HOXC8, HOXB8, ETV1, ETV5, T, PITX2, MITF, ZFHX4, HOXA9, IRF2, CNOT2, MYBL1, GNOT1, TEF, REL, TSHZ3, ELK3, ATF7IP, EP300, CREB3L2, GABPA, SIM2, ELF1, CBFA2T2, HNF4A, RERE, ETV7, STAT3, NCOA2, HOXA6, JARID2, ESRRG, HSF2, NCOA1, HDX, SLC29A3, HOXD4, HOXC9, RCJMB04_17o18, ALX1, LOC429524*
0030528	Transcription factor activity	0	*NR1H4, IRX2, SOX9, IRX1, HDAC4, FOXO1A, NR2F2, SMAD3, TCF3, BETA3, EBF3, NR1H3, TOB1, ID3, ID1, GBX1, CITED4, SOX8, TWIST1, HOXB3, RUNX1T1, PBX1, MEOX1, VSX1, SMAD9, SOX10, MEIS2, NKX6-2, MEF2A, NEUROG1, TFAP2B, NKX-6.1, ID2, PITX2, MAFA, MITF, ZFHX4, PAX7, PAX6, GBX2, NR5A2, TFAP2A, NFE2L2, ZEB1, MYBL1, PGR, RARB, TCF12, LHX1, NTN1, GATA5, TSHZ3, RCJMB04_6b10, DBX2, ATF7IP, EP300, GABPA, C21orf66, SIM2, TFDP1, ELF1, GATA3, HNF4A, HES5, NCOA2, RCJMB04_3c23, GTF3C6, DST, NCOA1, HDX, SLC29A3, LHX8, HES6, LASS5, TSHZ1, RCJMB04_8e18, FOXP2*

Table S5 Hox gene expression across the elongating neural axis
Significantly regulated probesets for all comparisons were assessed for differential Hox gene expression. The pooled SZ + PNT vs CNT + RNT comparison indicated that Hox gene paralogues between 4 and 9 are all detected in the SZ+PNT at higher levels than in the CNT+RNT, while HoxB3 is enriched in the CNT+RNT. Pairwise comparisons between the four rostro-caudal regions revealed higher expression of paralogues 6 to 9 in SZ in comparison with PNT, and higher 6 to 9 in PNT than in CNT, and that CNT expresses higher 4-7 paralogues than RNT. Note extensive shared expression of Hox genes paralogues across SZ, PNT and CNT and that cells in the SZ, PNT and CNT have yet to acquire a final Hox code [1]. Each shade of blue represents a different paralogous group.

Hox gene	SZ + PNT vs CNT + RNT		SZ vs PNT		PNT vs CNT		CNT vs RNT	
B3								
A4								
B4								
D4								
A6								
A7								
B7								
B8								
A9								
B9								
C9								

Hox gene paralogue group	SZ	PNT	CNT	RNT
3				
4				
6				
7				
9				

1 Deschamps, J. *et al.* Initiation, establishment and maintenance of Hox gene expression patterns in the mouse. *Int J Dev Biol* **43(7**, 635-650. (1999).

Table S6 KEGG pathway identification of t-RNA biogenesis, proteasome and cell cycle-associated genes

Genes that are upregulated green or downregulated red in the CNT+ RNT in comparison with the SZ+ PNT; in brackets, additional manual annotation of related genes from statistically significantly regulated gene lists.

KEGG Pathway ID	Description	p-value	Genes
03050	Proteasome	0.0274	*PSMA7, PSMD7, PSMB3, PSME3, PSME4, PSMB1, PSMD1, PSMD3*
00970	Aminoacyl-tRNA biosynthesis	0.0016	*KARS, IARS, HARS, RARS, LARS, YARS, EPRS, WARS, FARSB, TARS /RCJMB04_4k14*
04010	Cell cycle	0.1404	*HDAC1, CCND2, SMAD3, CCND1, CCNB2, RBL2, CDC45L, EP300, TFDP1, DBF4, CDK6, BUB1, ORC3L, MAD2L1, WEE1, ORC4L, CCNE1, GADD45A (CCNG1, CDC14A, CDKL2/CDK1, CENPJ, CKAP2,) (CDC37, CDK6, CUL3, DNM1L, RCJMB04_1o21/MOB1)*

Table S7
Notable GO terms assigned to genes significantly enriched in SZ+PNT or CNT+RNT in comparison with each other

SZ + PNT	
t-RNA metabolic processes	GO:0006399
Ribosomal RNA processing	GO:0008033
Actin filament depolymerisation	GO:0030042, GO: 00300837
Cell leading edge	GO:0031252
MAP kinase phosphatase activity	GO:0033549
Oxidoreductase activity	GO:0016614
Hormone binding	GO:0042562

CNT + RNT	
Cell size	GO:0008361
Cell growth	GO:0040007
Biological adhesion	GO:0022610
Neuron projection	GO:0043005
Membrane-bounded organelle	GO:0043227
Membrane receptor signalling	GO:0019199, GO:0005057
Cell surface	GO:0009986
